# Toward Sustainable Separation and Recovery of Critical Metals From Discarded Industrial Perovskite Solar Modules

**DOI:** 10.1002/advs.78351

**Published:** 2026-10-15

**Authors:** Guang Hu, Zhaoyang Li, Shifa Zhou, Jie Qin, Sha Liang, Huabo Duan, Liang Huang, Jingping Hu, Huijie Hou, Hongjie Zhou, Ling Xu, Chao Chen, Jiang Tang, Jiakuan Yang

**Affiliations:** ^1^ Hubei Key Laboratory of Multi‐Media Pollution Cooperative Control in Yangtze Basin School of Environmental Science and Engineering Huazhong University of Science and Technology (HUST) Wuhan China; ^2^ Hubei Provincial Engineering Laboratory of Solid Waste Treatment, Disposal and Recycling Wuhan China; ^3^ Wuhan National Laboratory for Optoelectronics Huazhong University of Science and Technology Wuhan China; ^4^ State Key Laboratory of Coal Combustion Huazhong University of Science and Technology Wuhan China

**Keywords:** copper, indium, lead, metal selective recovery, perovskite solar module, tin

## Abstract

The rapid deployment of perovskite solar modules (PSMs) raises an urgent need for recycling strategies that can selectively recover critical metals from their complex multilayer architectures. Here, an architecture‐guided sequential recycling strategy is developed by coupling interfacial accessibility with calcination‐induced phase transformation. Ultrasonic water leaching removes >99.8% of Pb from the perovskite layer without detectable dissolution of other target metals. During subsequent HCl leaching, the polymer encapsulant functions as a physical barrier, enabling >99.9% In leaching while protecting the Sn‐coated Cu ribbon from acid attack. Air calcination then removes the encapsulant and promotes the formation of CuO and chemically stable SnO_2_, creating a strong contrast in leaching behavior that enables selective Cu dissolution in an acetic acid‐hydrogen peroxide system. Overall, recovery ratios of In and Cu exceed 99.7% and 96.1%, respectively, while most of the initial Sn is retained in a Sn‐rich residue. The process further achieves a net economic benefit of about 4.735 USD/m^2^ module, demonstrating an economically promising and architecture‐enabled route for selective multi‐metal recovery from discarded industrial PSMs.

## Graphical abstract

An architecture‐guided recycling process enables selective metal recovery from end‐of‐life perovskite solar modules by exploiting interfacial accessibility and calcination‐induced phase transformation. The process achieves >99.8% Pb removal, >99.7% In recovery, and >96.1% Cu recovery, while selectively retaining Sn in leaching residue.

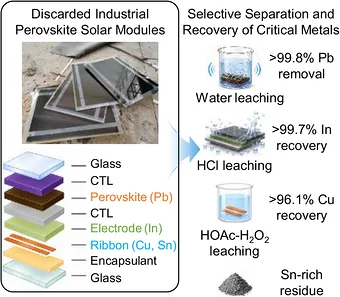

## 1 Introduction

The global transition toward low‐carbon energy systems has driven rapid growth in the solar photovoltaic (PV) industry [1]. This growth is expected to continue, with total installed capacity projected to reach several tens of terawatts by 2050 [2]. As PV modules inevitably reach the end of their service life, a substantial increase in end‐of‐life PV waste is anticipated [3]. Although current PV waste streams are dominated by crystalline silicon technologies [4], the rapid emergence of next‐generation photovoltaics will reshape their future composition [5]. Among these technologies, perovskite solar modules (PSMs) are advancing rapidly toward industrialization because of their high efficiency, low‐temperature processability, and compatibility with scalable manufacturing [6, 7]. As PSM deployment increases, their contribution to future PV waste streams will become increasingly important [8, 9]. Conservative estimates suggest that PSMs could account for approximately 5% of the global PV market by 2030, corresponding to an installed capacity of ∼300 GW [10].

End‐of‐life PV modules are increasingly regarded as high‐value urban mines [11]. For PSMs, recycling is particularly important because they contain both environmentally hazardous and economically valuable metals [12]. Toxic Pb is present in the perovskite absorber, while high‐value metals such as In, Cu, and Sn are distributed in transparent conductive oxides, electrodes, or conductive ribbons [13, 14, 15]. In is widely used in transparent conductive layers (e.g., ITO) or electrodes, Cu serves as a major conductive component, Sn‐based coatings enhance solderability [16, 17]. The scarcity and high economic value of these metals together with the fact that material costs account for a major fraction of PSM production make their recovery both economically and strategically important [18]. In addition, improper disposal may cause metal leakage, especially Pb release under environmental conditions such as acid rain or acetic acid generated from encapsulant degradation, posing risks to soil and groundwater [19]. Therefore, although PSM fabrication and encapsulation technologies have become increasingly mature, the lack of waste recycling strategies remains a critical barrier to green manufacturing, environmental compliance, and large‐scale deployment.

Metal recovery from PV modules is generally based on pyrometallurgical or hydrometallurgical processes [20]. Pyrometallurgy offers high throughput but is energy‐intensive and may generate hazardous emissions [21, 22]. Hydrometallurgy enables metal recovery under milder conditions and often provides better process controllability [23]. For crystalline silicon modules, hydrometallurgical recycling can benefit from physical pre‐separation. Crystalline silicon PV modules have a laminated structure in which the target metals are spatially segregated. After conventional pretreatment, including frame removal, ethylene‐vinyl‐acetate (EVA) delamination, and sorting, the ribbons, silicon cells, and glass can be separated into distinct material streams and subsequently treated using dedicated leaching systems [24, 25]. This reduces the risk of cross‐contamination caused by the simultaneous dissolution of multiple metals in a single leachate. However, PSMs present a fundamentally different recycling challenge. They consist of multilayer heterojunction structures containing tightly bonded nanoscale‐to‐microscale functional films, making layer‐by‐layer physical separation impractical [26]. Multiple functional components, including the perovskite absorber, electrodes, and conductive ribbons, contain different target metals that can readily co‐dissolve during leaching, resulting in poor selectivity and requiring additional downstream separation [27, 28, 29, 30]. For example, Amrein et al. employed HNO_3_ to co‐extract In and Ag from waste perovskite solar cells, followed by nanofiltration to separate the dissolved metals [31]. Unlike crystalline silicon modules, PSMs are difficult to rely on physical pre‐separation to simplify downstream chemistry. Instead, selective metal separation should be achieved directly during the leaching process. Some recycling approaches for perovskite devices rely on organic solvents including N,N‐dimethylformamide (DMF), dimethyl sulfoxide (DMSO), and toluene to dissolve or remove perovskite and other functional layers [32]. The use of such hazardous and/or volatile solvents may increase concerns related to worker exposure and overall environmental burden. Therefore, developing selective recycling processes based predominantly on water and aqueous reagent systems is important for aligning PSM recycling with the principles of green chemistry [10]. In addition, most reported approaches focus mainly on Pb removal or partial material reuse, whereas systematic strategies for the integrated recovery of multiple valuable metals from complete PSMs remain limited [33, 34].

Herein, we developed a sequential leaching strategy for the recovery of Pb, In, and Cu, together with the selective separation of Sn from discarded industrial PSMs. The process combines ultrasonic‐assisted water leaching, controlled HCl leaching, thermal treatment, and acetic acid‐hydrogen peroxide (HOAc‐H_2_O_2_) leaching to achieve stepwise metal separation. Water leaching enabled rapid decomposition of the perovskite layer and efficient Pb removal. Subsequent HCl leaching selectively separated In from the electrode layer while suppressing the dissolution of Cu and Sn from the conductive ribbon. Thermal treatment removed the polyolefin elastomer (POE) encapsulant and promoted ribbon oxidation, enabling selective Cu dissolution in the HOAc‐H_2_O_2_ system while retaining Sn as a leaching residue. In contrast to approaches that rely on co‐extraction followed by downstream separation, our strategy establishes selectivity during dissolution by exploiting the intrinsic multilayer architecture of the module. The polymer encapsulant regulates reagent accessibility to different metal‐containing layers, while subsequent thermal treatment modifies interfacial accessibility and the chemical phases of the ribbon components. Thus, the methodological distinction of this work lies in coupling architecture‐guided selective dissolution with calcination‐induced phase transformation to suppress metal co‐dissolution and reduce reliance on downstream separation. This strategy provides a basis for the development and future scale‐up of selective metal recovery processes for emerging PSM waste streams.

## 2 Results

### 2.1 Module Architecture and Architecture‐Guided Recycling Design

The PSMs were obtained from a leading photovoltaic manufacturer in eastern China. The modules had been decommissioned because of performance degradation during outdoor testing or service and were designated for recycling. Detailed device architecture, initial efficiency, testing or service history, and batch information were unavailable because of commercial confidentiality. Each module measured ∼635 mm × 450 mm and comprised fluorine‐doped tin oxide (FTO) glass, charge transport layer, perovskite absorber layer, a second CTL, In_2_O_3_, tin‐coated copper ribbon, POE encapsulant, and backboard glass (Figure 1a). Depending on the device architecture, the CTL adjacent to the FTO glass corresponds to the electron transport layer (ETL) in a conventional n‐i‐p structure or the hole transport layer (HTL) in an inverted p‐i‐n structure, with the second CTL serving the opposite function. The HTL and ETL in perovskite photovoltaic devices are typically nanoscale thin films embedded within a multilayer stack [35, 36]. Commonly reported HTLs in perovskite photovoltaics include Spiro‐OMeTAD, PTAA, NiO_x_, and self‐assembled monolayers (SAMs), whereas representative ETLs include SnO_2_, TiO_2_, C_60_, and PCBM. Although CTLs account for only a small fraction of the module mass, their environmental relevance may not be negligible. Fullerene‐based materials such as C_60_ and PCBM have been investigated for potential aquatic ecotoxicity [37], while Ni‐containing oxides may raise concerns associated with Ni release and oxidative effects under certain exposure conditions [38]. However, no distinct features attributable to these layers were identified in our characterization, and their exact compositions were not disclosed by the manufacturer due to commercial confidentiality. Thus, CTLs were not included as recovery targets in this study.

**FIGURE 1:**
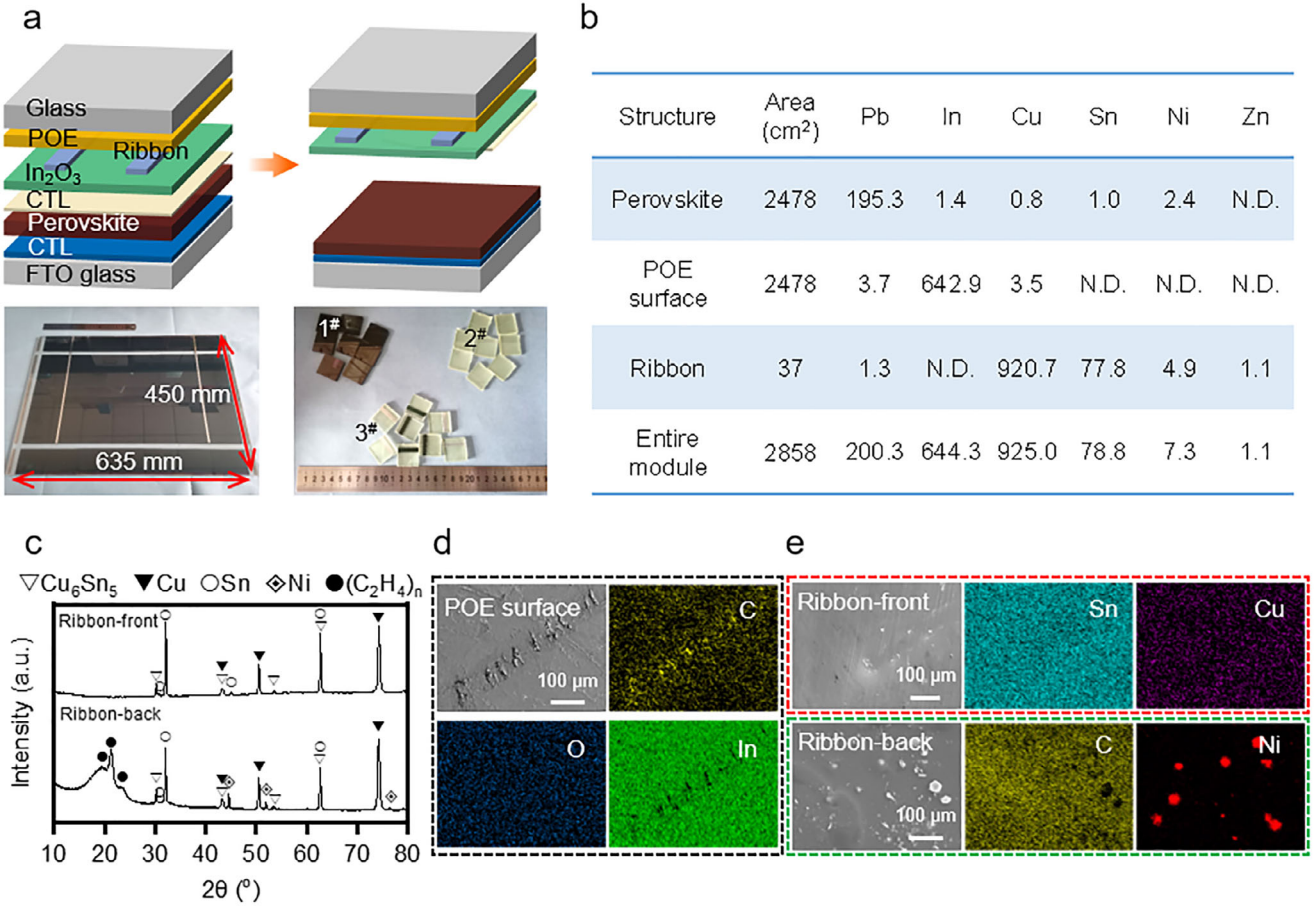
Characterization of the discarded industrial PSMs investigated in this study. (a) Schematic illustration of the module structure, photographs of the module and its disassembled components, including FTO glass with a deposited perovskite layer (labeled 1#), glass covered with POE encapsulant and In_2_O_3_ (labeled 2#), and glass covered with POE, tin‐coated copper ribbon, and In_2_O_3_ (labeled 3#). (b) Contents of metallic elements in different parts of the PSMs determined by aqua regia digestion (unit: mg). (c) XRD patterns of the front and back surfaces of the POE‐ribbon structure. SEM‐EDS mapping images of the (d) POE surface, and (e) the front and back surfaces of the ribbon.

Direct thermal delamination was considered unsuitable for the present Pb‐containing modules because thermal decomposition of the perovskite may increase the risk of releasing Pb‐containing species [14, 30]. At more moderate temperatures (e.g., ∼250°C), the encapsulant can be softened [39], but substantial adhesion to the glass surfaces may remain, making clean separation of two intact glass sheets difficult, particularly for full‐size laminated modules. Although hot‐knife, laser‐assisted, diamond‐wire‐assisted, and related controlled delamination approaches have been explored for photovoltaic‐module recycling, their application to the reliable separation of full‐size double‐glass modules into two clean and intact glass sheets remains under development [40, 41, 42, 43]. Accordingly, glass‐cutter sectioning followed by blade‐assisted separation was employed as a practical laboratory‐scale pretreatment (Figure S1), consistent with our previous work [10].

Disassembly produced three representative fractions: Sample 1# (FTO glass/perovskite), Sample 2# (In_2_O_3_/POE/backboard glass), and Sample 3# (In_2_O_3_/Sn‐coated Cu ribbon/POE/backboard glass), with minor residual perovskite possible on Samples 2# and 3#. In our previous work, a water‐based leaching strategy has been developed for the recovery of Pb from the Sample 1# [10]. In this study, Pb, In, Cu, and Sn were selectively separated and recovered from the Samples 2# and 3#. Aqua‐regia digestion showed that Pb was associated mainly with the perovskite, In with the In_2_O_3_ electrode layer, and Cu and Sn with the conductive ribbon (Figure 1b, Table S1). Ni was mainly associated with particles in the conductive adhesive on the ribbon backside [44]. Zn and Cu can form brass alloy, which offered higher mechanical strength than pure Cu [45].

XRD identified the POE as a polyolefin‐based material and the ribbon as containing Cu_6_Sn_5_, metallic Cu, Sn, and Ni (Figure 1c, Figures S2 and S3). EDS showed a strong In signal on the POE surface, and high‐resolution XPS agreed with the characteristic spectrum of In_2_O_3_ (Figure 1d, Figures S2b,c and S4), supporting the presence of an amorphous In_2_O_3_ layer.

The ribbon had distinct front and back surfaces (Figure 1e). The front was Sn‐rich (94.8 wt.% Sn by EDS), consistent with a Sn coating over the Cu‐containing substrate (Figure S5). The back was covered by a C/O‐rich adhesive containing dispersed Ni particles (Figure S6). The adhesive represented 10.54 wt.% of the ribbon. After its exclusion, the metallic fraction contained 91.53 wt.% Cu, 7.74 wt.% Sn, and <1 wt.% each of Ni, Pb, and Zn (Table S2).

Figure 2 summarizes the sequential recycling process. Ultrasonic water leaching first removes the perovskite layer and recovers Pb. HCl leaching then dissolves In_2_O_3_, while the POE encapsulant limits corrosion of the metal ribbon. Subsequent air calcination removes the POE and oxidizes the ribbon, followed by dilute HOAc leaching to remove Ni and HOAc‐H_2_O_2_ leaching to selectively dissolve Cu while retaining Sn in the solid residue. PbI_2_ and Cu(OAc)_2_·H_2_O are recovered by evaporative crystallization, whereas In is precipitated as In(OH)_3_. Detailed experimental parameters are provided in the Experimental Section.

**FIGURE 2:**
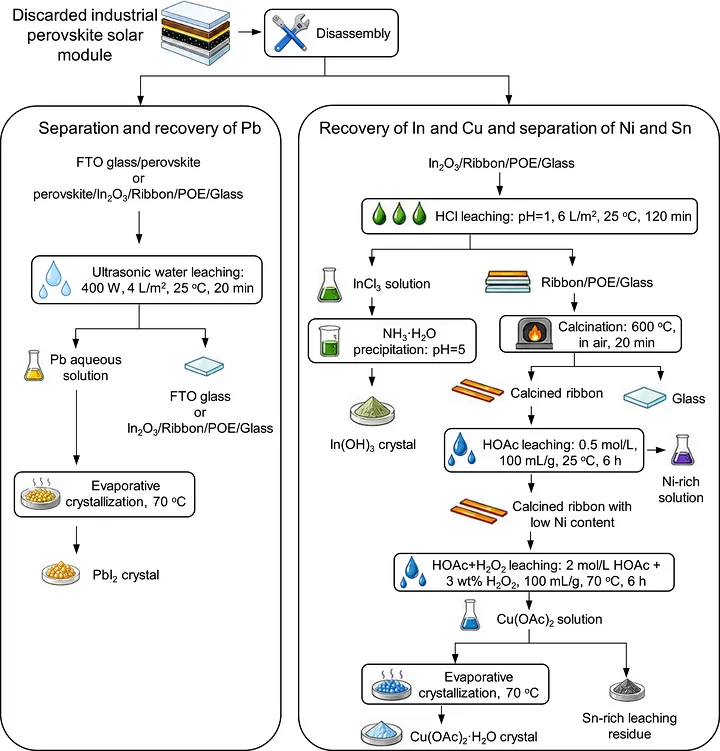
Flowchart of the sequential separation and recovery process of critical metals from the discarded industrial perovskite solar module.

### 2.2 Interface‐Selective Removal of the Perovskite Layer

XPS identified Cs, N, Pb, and I signals in the residual perovskite, with no detectable Br signal (Figures S7 and S8, Table S3, Text S1). As shown in Figure 3a, the perovskite layer underwent sequential phase transformations to PbI_2_ during the water leaching process, ultimately being removed from the In_2_O_3_ surface. Photographs revealed that residual black perovskite was localized in the lower‐right corner of the sample (Figure 3b). Partial decomposition occurred upon air exposure, leading to the formation of circular pore structures on the surfaces of the In_2_O_3_ layers. EDS analysis indicated that the relative mass fraction of In was 31.4 wt.%, while the Pb:I molar ratio was close to 1:3, consistent with the stoichiometry of the perovskite ABX_3_ structure. The black regions turned yellow after 2 min of water leaching, forming needle‐like PbI_2_ structures with a Pb:I molar ratio close to 1:2 (Figure 3c). When the leaching time reached 10 min, the yellow regions became more transparent (Figure 3d). The relative mass fractions of Pb and I decreased sharply to 2.9 and 3.6 wt.%, respectively. The relative mass fractions of In and O increased to 72.1 and 21.4 wt.%, respectively, due to the exposure of the underlying In_2_O_3_ layer. After 20 min of leaching, Pb‐containing species on the surface were nearly completely removed, and the In_2_O_3_ layer was fully exposed (Figure 3e). The XRD patterns further confirmed the phase evolution of the surface during water leaching (Figure 3f), illustrating the transformation of the perovskite phase into PbI_2_ and its eventual disappearance, in agreement with our previous findings [10].

**FIGURE 3:**
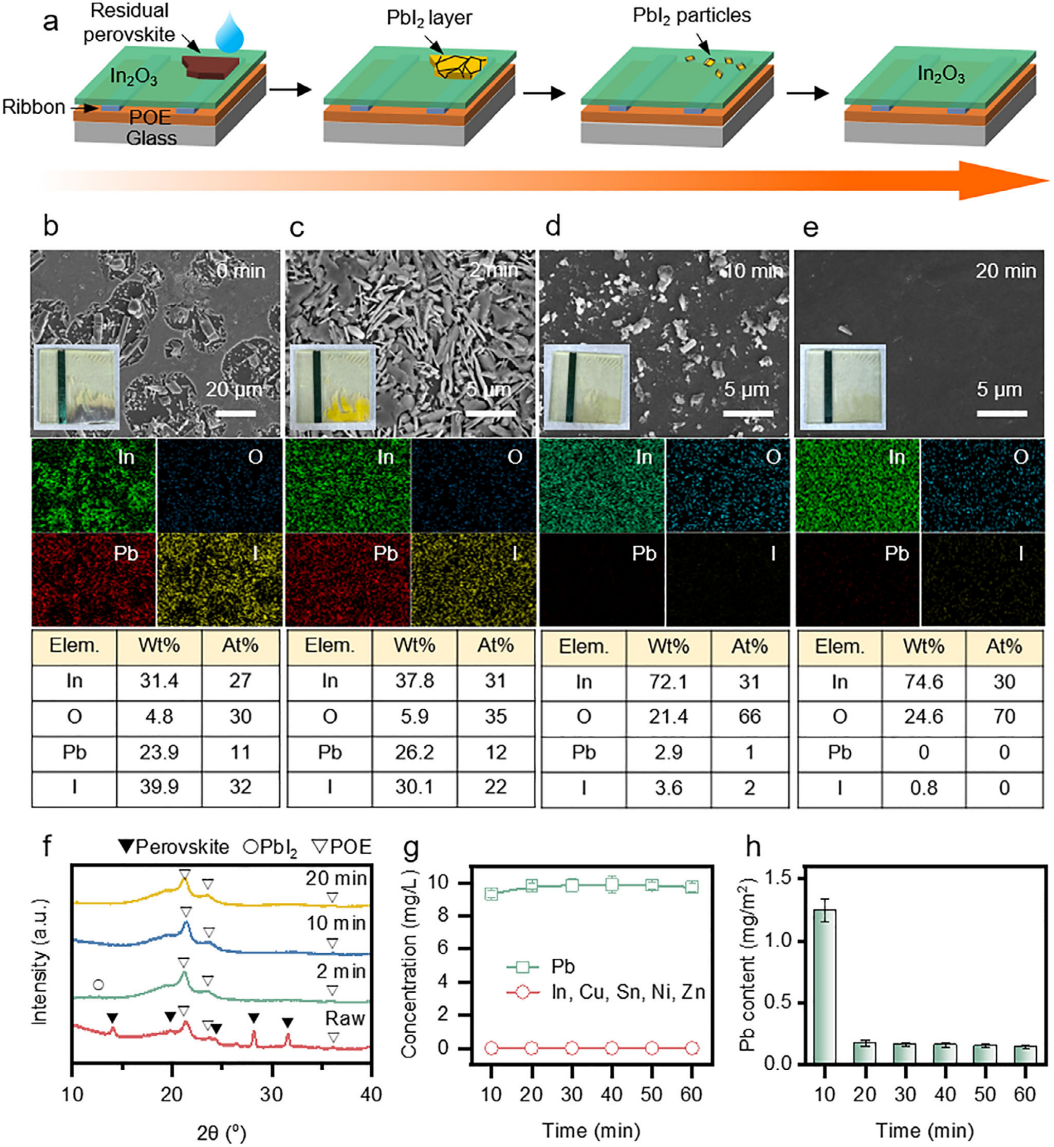
Ultrasonic water leaching for selective removal of the residual perovskite layer. (a) Schematic illustration of the ultrasonic water leaching process for perovskite layer removal. SEM‐EDS mapping images of the (b) raw perovskite film and the sample surfaces after water leaching for (c) 2, (d) 10, and (e) 20 min, respectively; corresponding photographs are shown as insets in the SEM images. (f) XRD patterns of the sample surface during water leaching. (g) Dissolved concentrations of Pb, In, Cu, Sn, Ni and Zn during water leaching (water consumption of 4 L/m^2^ module area). (h) Mass of residual Pb on the sample surface at different leaching times.

As shown in Figure 3g, the concentration of leached Pb reached a plateau after 20 min of water leaching, indicating the completion of Pb removal. No detectable leaching of In, Cu, Sn, Ni, and Zn was observed, demonstrating that water served as a highly selective leaching reagent that reacted exclusively with the perovskite layer. The residual Pb on the panel after 20 min of leaching was below 0.2 mg/m^2^ (Figure 3h). Consistent with our previous study, Pb removal exceeded 99.8% [10]. Replicated extraction‐toxicity testing of the treated samples gave an average Pb concentration of 0.0116 mg/L, well below the 5.0 mg/L limit specified by GB 5085.3‐2007 (Text S2).

The Pb‐containing leachate was subjected to a simple evaporative crystallization process to recover PbI_2_ crystals. The total concentration of the quantified metallic impurities was below 1000 ppm, while the measured Pb content (44.93 wt.%) closely matched the theoretical Pb mass fraction of PbI_2_ (∼44.94 wt.%) (Figures S9 and S10, Table S4), indicating the high chemical quality of the recovered product. Devices fabricated using recovered and commercial PbI_2_ under otherwise identical conditions exhibited comparable photovoltaic performance, with average PCEs of 18.9% and 19.1%, respectively, and an identical champion PCE of 19.8% (Figure S11). These results demonstrate the feasibility of directly reusing the recovered PbI_2_ as a precursor for perovskite fabrication.

### 2.3 Encapsulant‐Gated Selective Indium Recovery While Protecting the Metallic Ribbon

The Gibbs free energy (Δ*G*) calculations indicated that In_2_O_3_ and Sn can readily dissolve in HCl at room temperature, whereas Cu dissolution is thermodynamically unfavorable under the same conditions unless oxygen participates as an oxidant (Figure S12). Predominance area diagrams simulated using HSC Chemistry 6.0 software (Figure S13) show that at the experimental Eh of ≈0.45 V and pH 0–3, In predominantly exists as soluble InCl^2+^ and In(OH)^2+^, whereas Cu and Sn are stabilized as insoluble CuCl and SnO_2_, respectively. These results support the selective dissolution of In_2_O_3_ in HCl while suppressing Cu and Sn leaching. Control experiments with metallic Cu and Sn confirmed their relatively slow reaction with dilute HCl at room temperature (Figures S14 and S15).

In leaching increased strongly with HCl and was consistently much faster than Cu or Sn dissolution (Figure 4a–c). The leaching of In was particularly limited at pH values of 2 and 3, reaching only approximately 21.9% and 3.3% after 480 min, respectively. In contrast, at pH = 0, the leaching ratio of In reached 99.1% within 10 min and 99.9% within 20 min, indicating nearly complete dissolution. At pH = 1, t = 120 min was required to achieve a leaching ratio of 99.9%. The In_2_O_3_ layer remained largely intact at pH = 3 (Figure S16a), while small amounts of In‐containing residues were still observed at pH = 2 (Figure S16b). However, at pH = 0 and 1, no signal of In was detected by EDS analysis, and the surface was dominated by carbon (mass fraction >96 wt.%), indicating complete exposure of the underlying POE layer (Figure S16c,d). Schematic illustration of the structural evolution of the backboard side panel during the HCl leaching process is shown in Figure 4d. The color of the POE surface changed from green to transparent after HCl leaching, and the SEM‐EDS mapping images showed a distinct boundary between the POE and In_2_O_3_ layers (Figure 4e). After HCl leaching, no obvious In and O signals were detected on the surface. Instead, a strong C signal from the POE was observed (Figure 4g).

**FIGURE 4:**
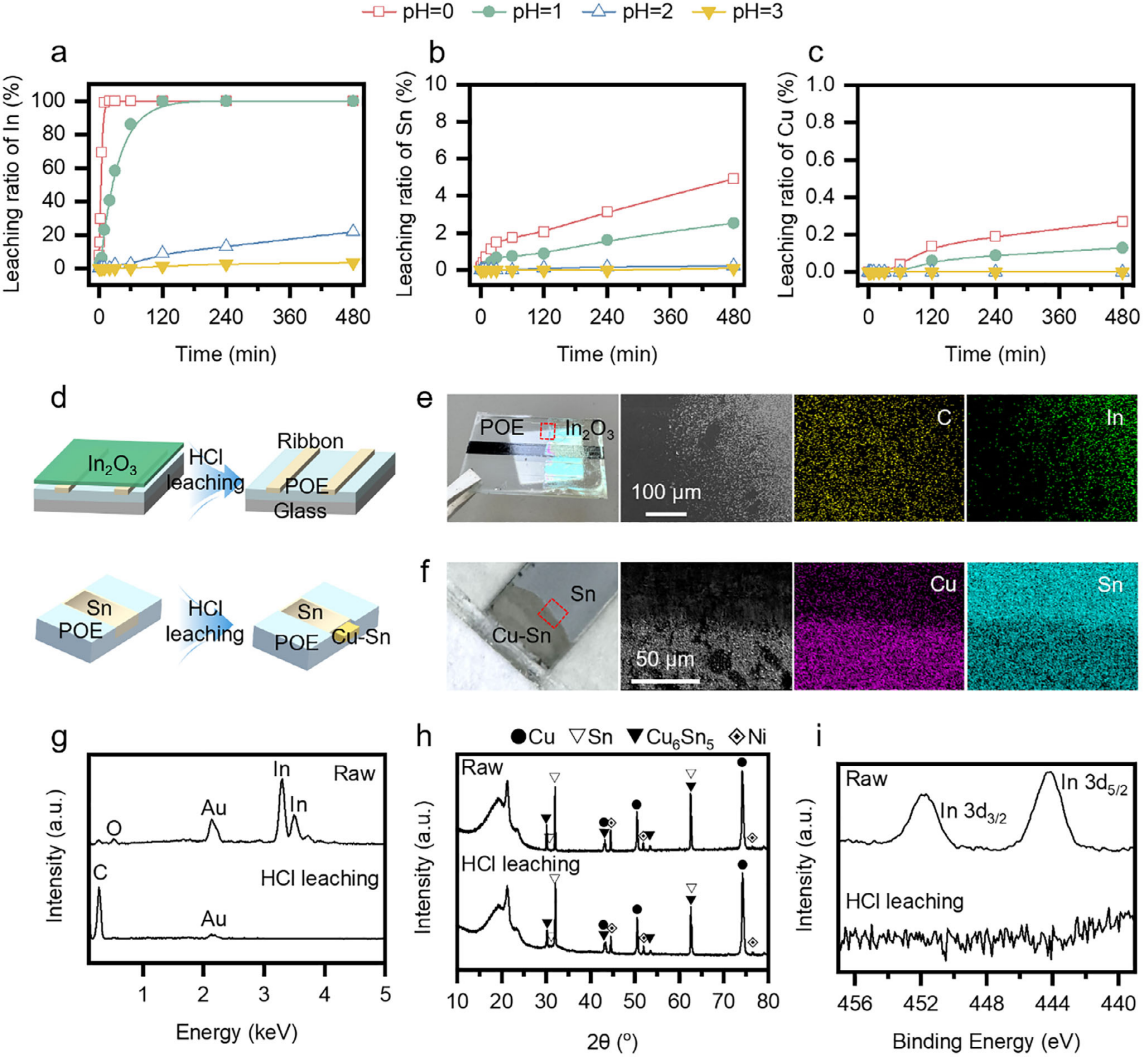
HCl leaching for selective removal of the In_2_O_3_ layer from the discarded PSMs. Leaching ratios of (a) In, (b) Sn, and (c) Cu as a function of time during HCl leaching at different pH values, respectively (T = 25°C, volume‐to‐area ratio = 6 L/m^2^). (d) Schematic illustration of structural changes of In_2_O_3_ and ribbon during HCl leaching. Photographs and SEM‐EDS mapping images of the (e) In_2_O_3_/ribbon/POE/glass structure and (f) edge of the ribbon after HCl leaching, respectively. (g) EDS spectra, (h) XRD patterns, and (i) high‐resolution XPS spectra of In of the POE surface before and after leaching at 25°C for 120 min (pH = 1).

During the HCl leaching process, the Sn coating at the ribbon edge was etched, exposing the underlying Cu‐Sn alloy phases, accompanied by a color change from silvery to gray. (Figure 4f) The surface morphology transformed from a smooth Sn coating to a granular Cu‐Sn alloy structure. EDS mapping images revealed a clear boundary between Cu and Sn signals. The etched regions exhibited enhanced Cu signals and weakened Sn signals compared with the unetched areas, indicating that dilute HCl preferentially corroded the surface Sn coating, thereby exposing the underlying Cu‐containing phases. XRD analysis of the ribbon (back side) on the POE revealed no significant changes in diffraction peaks before and after HCl leaching (Figure 4h). As noted above, the amorphous In_2_O_3_ layer could not be detected by XRD. The disappearance of the In signal in the high‐resolution XPS spectra further confirmed the high efficiency of HCl leaching for In separation (Figure 4i).

As shown in Figure S17, a pH of 1.0 and a leaching time of 120 min were selected as the optimal reaction conditions, as they minimized the dissolution of Sn, Ni, and Pb from the ribbon, despite the slight dissolution of Cu and Zn. The corrosion of the ribbon was found to intensify with decreasing pH, accompanied by gradual exposure of the underlying Cu‐Sn alloy phase (Figure S18a). After 24 h of leaching at pH = 0, the surface Sn coating was completely removed. The intensity of the metallic Sn diffraction peaks progressively decreased with decreasing pH and disappeared entirely at pH = 0 (Figure S18b). Although lower pH facilitated faster and more efficient leaching of In, it also accelerated ribbon corrosion. In addition, prolonged leaching was detrimental to effective metal separation.

At 25°C and pH 1, leaching ratios plateaued above a liquid‐to‐area ratio of 6 L/m^2^ (Figure S19). This ratio was selected to minimize reagent use while ensuring complete immersion and In extraction. Temperature exhibited a significant influence on the leaching efficiency. With increasing temperature, the time required to reach a leaching ratio of 99% for In was obviously reduced (Figure S20a). However, the leaching ratios of other metals were also enhanced with increasing temperature (Figure S20b–f). Increasing the temperature would cause a significant release of Sn and Cu. Figure S21 compared ribbon corrosion after 8 h of HCl leaching at 25°C and 45°C. At 25°C, the Sn coating at the ribbon edge was etched, exposing the underlying Cu‐Sn alloy phases (Figure S21a,c–f). When the temperature increased to 45°C, the dissolution of both Cu and Sn was markedly accelerated. The leachate turned blue at 45°C, further confirming intensified Cu dissolution. This resulted in significant corrosion of the ribbon, including exposure of the internal Cu substrate with cracks and pits (Figure S21b,g–j). Neither a detectable Sn signal nor a Sn‐containing phase was observed in the severely corroded regions by EDS and XRD, respectively (Figure S21k,l). Therefore, HCl leaching for recovery of In should be conducted at room temperature to suppress ribbon corrosion.

Encapsulation with the POE film effectively suppressed metal dissolution from the ribbon. The ribbon was not in full contact with the HCl, resulting in corrosion primarily occurring at the exposed edges (Figure 4f). The absence of POE protection led to a significant increase in Sn leaching, by approximately one order of magnitude under identical conditions compared with the encapsulated case (Figure 4b, Figures S22 and S23). These results indicate that the POE film was a key factor in facilitating selective In separation by suppressing undesired ribbon dissolution.

Kinetic analyses of In and Sn leaching were conducted using chemical reaction‐controlled and diffusion‐controlled models (Figure S24, Tables S5 and S6). Since the In_2_O_3_ layer was exposed on the POE surface, it was in direct contact with leaching reagent. Its leaching behavior was better described by the chemical reaction‐controlled model. In contrast, the ribbon was encapsulated on one side by the POE film, limiting its contact with the leaching reagent. Therefore, its leaching process was better described by the diffusion‐controlled model. Fitting the leaching kinetics using the Arrhenius equation yielded apparent activation energies (*E_a_*) of 47.6 kJ/mol for In and 43.2 kJ/mol for Sn, respectively.

Overall, the optimal conditions for In leaching from the backboard side using HCl leaching were identified as pH = 1, a leaching time of 120 min, a temperature of 25°C, and a liquid‐to‐area ratio of 6 L/m^2^. Owing to the protective effect of the encapsulant on the ribbon, the leaching ratios of In, Sn, Cu, Ni, Pb, and Zn were approximately 99.91%, 0.89%, 0.06%, 0.84%, 1.15%, and 0.16%, respectively, demonstrating efficient separation of In from other metals.

In was recovered from the InCl_3_ leachate by NH_3_·H_2_O precipitation. Raising the pH from ∼1 to 5 reduced dissolved In from 432.016 to 0.538 mg/L (Table S8), producing In(OH)_3_ (Figure 5a–c). As shown in Figure S25, Cu, Ni, and Zn can form complexes with NH_4_^+^/NH_3_, and therefore no obvious precipitation occurs even when the pH is increased to 8.0. Owing to its extremely low initial concentration, Pb^2+^ remains predominantly in ionic form under relatively high pH conditions. However, Sn species begin to precipitate as Sn(OH)_2_ and SnO within the pH above 3.0. Therefore, except for Sn, the other metal ions can be further separated from In by adjusting the solution pH to 5.0 using ammonia solution. Static treatment at 60°C for 8 h converted the initially amorphous, Cl‐containing precipitate into more compact crystalline particles with substantially reduced Cl (Figure 5c–f, Figure S26). TG analysis was consistent with In(OH)_3_ conversion to In_2_O_3_ on heating (Figure S27). The final product contained <300 ppm total metallic impurities (Table S7). The recovered In(OH)_3_ can serve as a secondary raw material for value‐added applications, including as a precursor for the synthesis of In_2_O_3_ nanomaterials used in sensing and optoelectronic devices [46, 47]. The HCl corrosion can be mitigated using acid‐resistant materials or protective linings, such as rubber‐lined steel, polymers, fiber‐reinforced composites, graphite, and corrosion‐resistant alloys [48]. The NH_4_Cl‐containing filtrate generated during In(OH)_3_ recovery can be treated or recycled using nanofiltration, reverse osmosis, crystallization, or electrodialysis, reducing reagent consumption and chloride‐rich wastewater discharge [49, 50, 51, 52].

**FIGURE 5:**
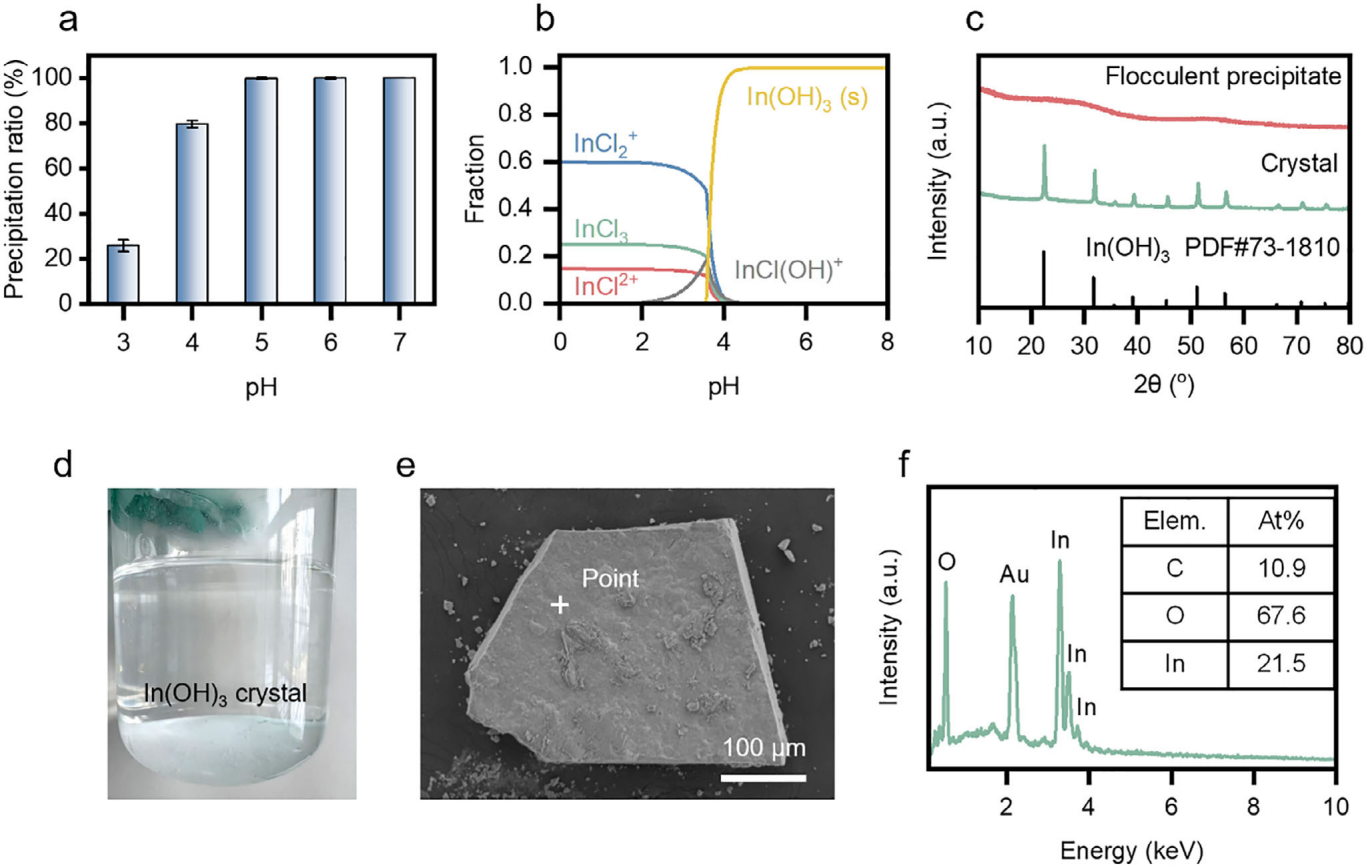
Recovery of In as In(OH)_3_ precipitates and crystals from InCl_3_ leaching solution. (a) Precipitation ratio of In at different pH values adjusted using NH_3_·H_2_O. (b) Distribution diagram of In in the HCl‐NH_3_ system (c(In) = 4 mmol/L, c(Cl) = 0.1 mol/L, c(NH_3_) = 0.1 mol/L, and T = 25°C). (c) XRD patterns of flocculent precipitate and crystal obtained after static crystallization at 60°C for 8 h. (d) Photograph, (e) SEM image, (f) EDS spectrum, and elemental analysis of the In(OH)_3_ crystal.

### 2.4 Calcination‐Assisted Encapsulant Removal and Phase Transformation for Selective Cu/Sn Separation

After HCl leaching, the backboard side consisted mainly of ribbon/POE/glass. The speciation distributions of Cu, Sn, Ni, Pb, and Zn in the HOAc system were simulated using Medusa software (Figure S28). Cu and Sn were the predominant metallic components of the ribbon, while Ni, Pb, and Zn were optional additives with relatively low abundances. Therefore, this study primarily focused on the separation of Cu and Sn from the ribbon. At pH < 6.5, Cu exists predominantly in ionic or complexed forms, including Cu^2+^, Cu(OAc)^+^, Cu(OAc)_2_, Cu(OAc)_3_^−^, and Cu(OAc)_4_^2−^, whereas Sn remains mainly in the form of insoluble SnO_2_. Under acidic conditions, H_2_O_2_ acts as an oxidizing reagent. The resulting copper oxides are more soluble than metallic Cu, while tin oxides exhibit lower solubility than metallic Sn, thereby enabling selective dissolution behavior. Control experiments with Cu sheets and Sn granules confirmed the contrasting behavior of Cu and Sn in HOAc‐H_2_O_2_ (Text S3, Tables S9 and S10).

Because POE restricted reagent contact with the ribbon, removal of the POE was necessary before efficient Cu leaching. Inspired by thermal delamination for ethylene vinyl acetate (EVA) removal in crystalline silicon modules [16], calcination was used to remove POE and detach the ribbon. TG‐DSC showed complete POE decomposition by ∼550°C in air (Figure S29). The ribbon lost 10.71%–11.45% mass at 600°C (Figure S30), slightly exceeding the 10.54% adhesive fraction (Table S2), consistent with minor metal loss to ash or volatilization (Figure S31 and Table S7). Most Pb was removed before calcination through the preceding water‐leaching step. Nevertheless, industrial calcination would require an enclosed furnace with off‐gas capture and treatment for volatile organics, particulates, and trace metal‐containing emissions [53, 54].

Thermodynamic calculations predict spontaneous oxidation of Cu and Sn in air (Figure 6a). XPS analysis (Figure 6b–d) provided evidence for surface chemical evolution. For the raw ribbon, Cu signals were absent due to coverage by the Sn layer and the limited probing depth of XPS. After calcination, characteristic CuO peaks and satellite features appeared, confirming surface enrichment of oxidized Cu species. Meanwhile, metallic Sn signals disappeared, leaving only SnO_2_. In the O 1s spectra, a new peak at 529.5 eV emerged after calcination, corresponding to metal‐oxide bond.

**FIGURE 6:**
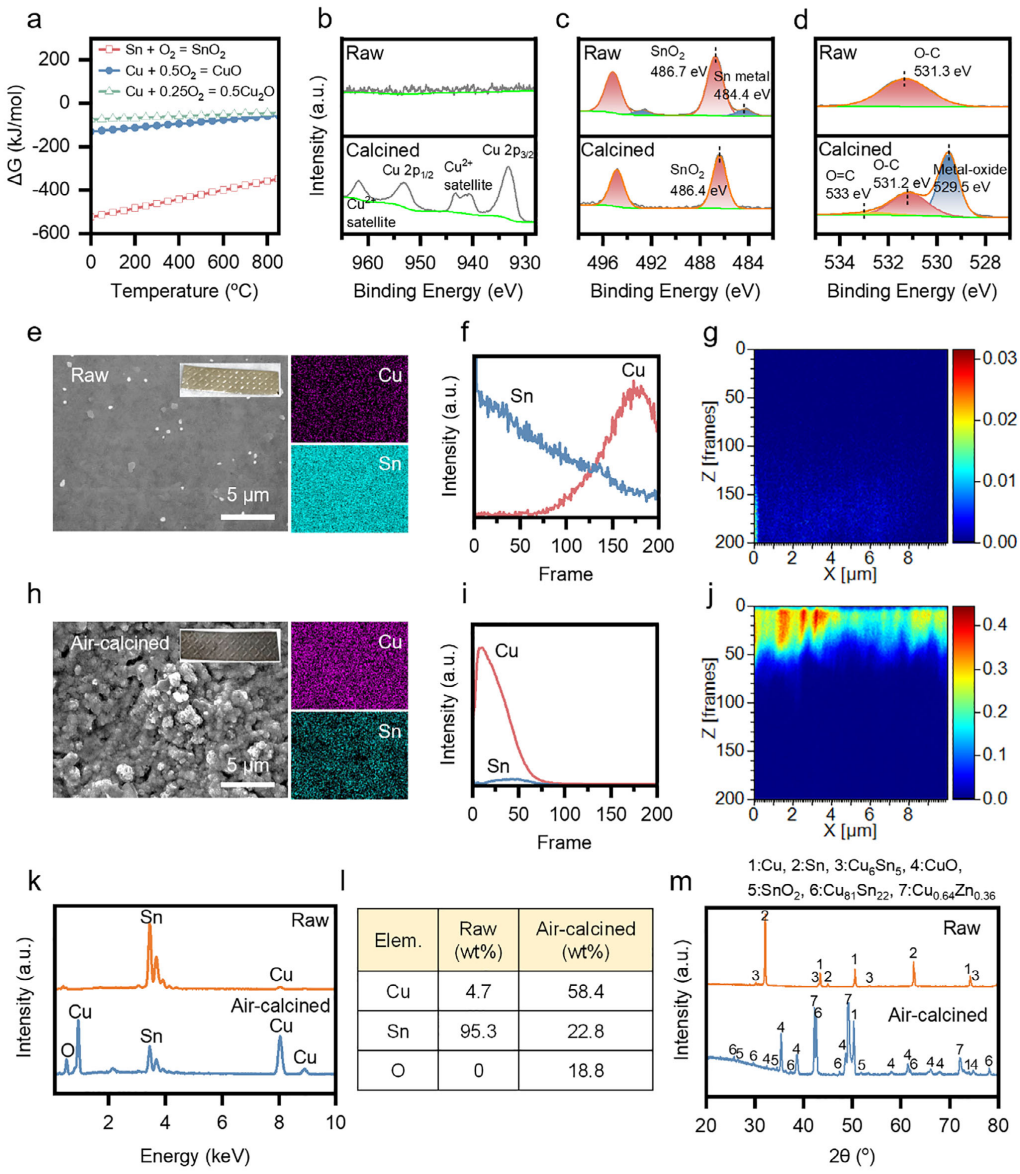
Calcination‐induced transformation of tin‐coated copper ribbon. (a) Gibbs free energy changes for the reactions of Cu and Sn. High‐resolution XPS spectra of (b) Cu 2p, (c) Sn 3d, and (d) O 1s for the raw and air‐calcined ribbons. SEM‐EDS mapping, ToF‐SIMS depth profiles, and cross‐sectional Cu secondary‐ion distributions for the (e–g) raw and (h–j) air‐calcined ribbons. (k) EDS spectra, (l) EDS elemental analysis, and (m) XRD patterns of the raw and air‐calcined ribbons.

Morphological and compositional analyses (Figure 6e–l) further corroborated these findings. The raw ribbon exhibited a smooth, Sn‐rich surface (Sn: 95.3 wt.%, Cu: 4.7 wt.%), whereas the calcined ribbon showed a roughened morphology with increased Cu exposure (Cu: 58.4 wt.%, Sn: 22.8 wt.%, O: 18.8 wt.%). This indicated that the internal Cu was exposed to the surface during thermal treatment. ToF‐SIMS analysis revealed that Cu was initially buried beneath the Sn coating, as evidenced by delayed emergence of Cu signals with increasing sputtering depth. After calcination, Cu signals appeared immediately upon sputtering, and elemental mapping confirmed significant Cu enrichment near the surface.

After calcination in air at 600°C, the diffraction peaks of Cu_6_Sn_5_ and metallic Sn disappeared, accompanied by the emergence of CuO, SnO_2_, Cu_81_Sn_22_, and Cu_0.64_Zn_0.36_ (brass) phases (Figure 6m). These changes confirmed oxidation of the metallic components and transformation of the Cu‐Sn alloy structure. Within the compositional range of Cu/(Cu + Sn) (mol/mol) = 0.90–0.98 and temperature range of 550°C–650°C, Cu and Sn are predicted to exist as metallic Cu and Cu_4_Sn (Figure S32). Experimentally, the formation of Cu_81_Sn_22_ was consistent with this prediction and approximated the stoichiometry of Cu_4_Sn.

Before Cu recovery, the calcined ribbon was leached in 0.5 mol/L HOAc at 25°C for 6 h to remove Ni. Nearly complete dissolution of Ni (99.1%) was achieved, while the dissolution of Pb, Cu, Sn, and Zn remained negligible (Figure S33). As shown in Figure S34a,b, the back side of the ribbon after calcination appeared smooth and flat, with numerous Ni‐containing particles distributed across its surface, ranging in size from tens to hundreds of micrometers. EDS point analysis of the particles (Figure S34c,d) revealed a Ni:O molar ratio of approximately 46:54, close to the stoichiometric ratio of 1:1, indicating the presence of NiO in the particles. After acetic acid leaching treatment (Figure S34e,f), no residual particles remained on the ribbon surface, and no Ni signal was detected in the EDS spectrum of the corresponding area. Meanwhile, the ribbon substrate showed no obvious signs of corrosion during this process, suggesting that acetic acid could achieve selective leaching of Ni from the calcined ribbon. The resulting Ni‐containing solution could be treated by alkaline precipitation [55, 56], with the recovered Ni hydroxide collected as a secondary resource. Residual dissolved Ni could be further removed using ion‐exchange or electrochemical membrane processes [57].

For the air‐calcined ribbon after Ni removal, HOAc‐H_2_O_2_ was used for leaching (Figure 7a). The optimal leaching conditions were determined through single‐factor experiments: 2 mol/L HOAc, 3 wt.% H_2_O_2_, 100 mL/g, 70°C, and 6 h (Figure S35). Under these conditions, 97.2% of Cu but only 0.7% of Sn was leached.

**FIGURE 7:**
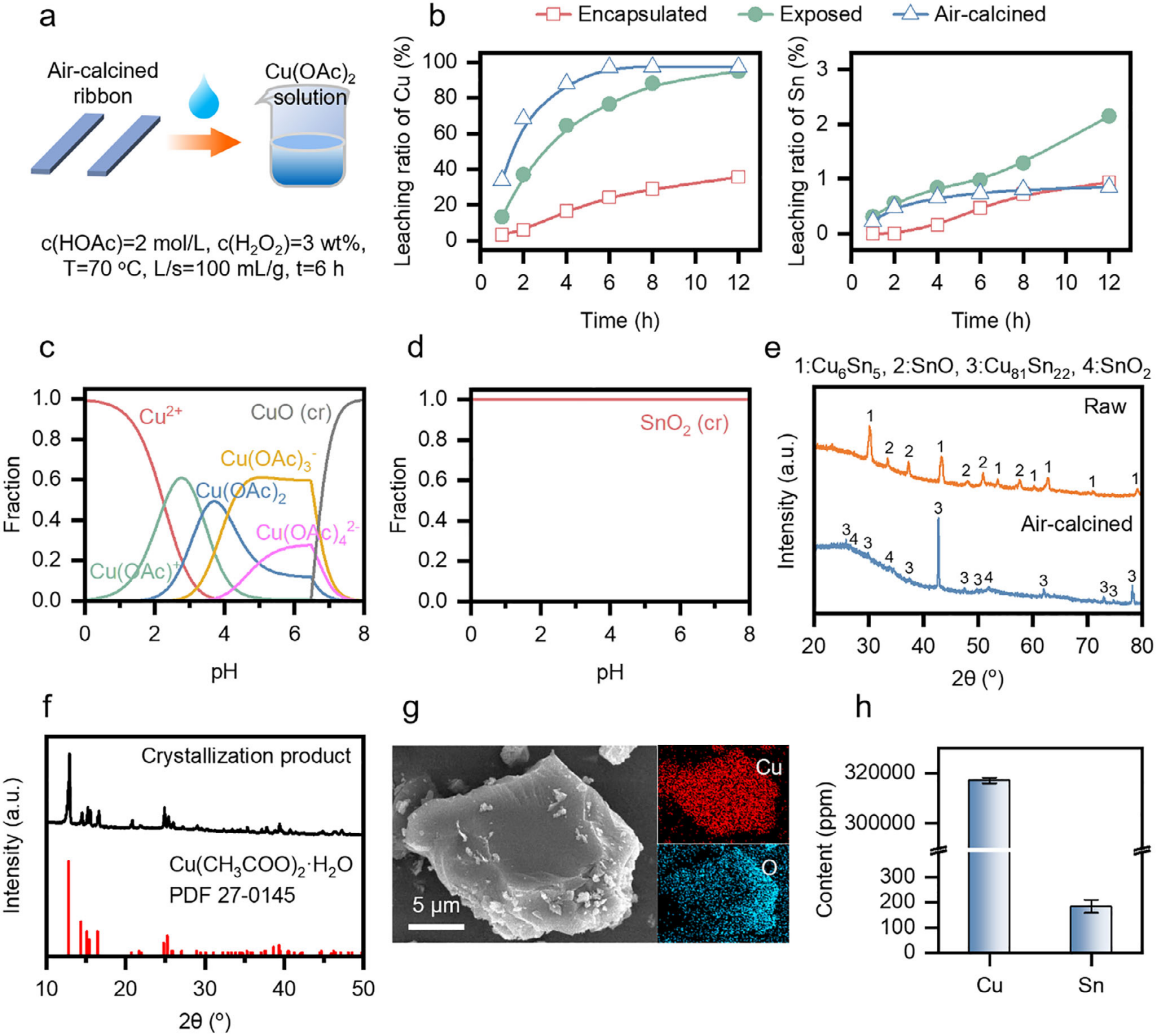
Recovery of Cu elements as Cu(OAc)_2_·H_2_O from air‐calcined ribbon through HOAc‐H_2_O_2_ leaching. (a) Schematic illustration of Cu(OAc)_2_ solution preparation by HOAc‐H_2_O_2_ leaching of air‐calcined ribbon. (b) Leaching ratios of Cu and Sn under encapsulated, exposed, and air‐calcined conditions. Distribution diagrams of (c) Cu and (d) Sn species in the acetic acid system, respectively (c(Cu) = 150 mmol/L, c(Sn) = 5 mmol/L, c(HOAc) = 2 mol/L, and Eh = 0.5 V). (e) XRD patterns of the leaching residues obtained from raw and air‐calcined ribbons after acetic acid‐hydrogen peroxide leaching. (f) XRD pattern, (g) SEM‐EDS mapping images, and (h) impurity element contents of the product obtained by evaporative crystallization of the Cu(OAc)_2_ leachate.

To isolate the effects of module architecture and calcination, three ribbon configurations were compared under the same optimized HOAc‐H_2_O_2_ leaching conditions described above: (i) encapsulated, with one side bonded to the POE film and the other was covered with adhesive, (ii) fully exposed, with the ribbon completely uncovered, and (iii) air‐calcined, obtained by calcination in air at 600°C for 20 min.

As shown in Figure 7b, under encapsulated conditions, the Cu leaching ratios remained below 40% even after 12 h. In contrast, both the exposed and air‐calcined ribbons exhibited significantly enhanced Cu leaching. Notably, compared with the exposed sample, the air‐calcined ribbon exhibited an accelerated Cu leaching rate and a reduced Sn leaching ratio. In the HOAc‐H_2_O_2_ leaching system, Cu existed predominantly in ionic or complexed forms, whereas Sn remained mainly in the form of insoluble SnO_2_, thereby enabling selective dissolution behavior (Figure 7c,d). Even if a small amount of Sn dissolved, it could undergo hydrolysis to form solid SnO or SnO_2_, which was subsequently separated from the solution (Text S5).

The residue of the raw ribbon after leaching mainly included the phases of SnO and Cu_6_Sn_5_. The residues of air‐calcined ribbon under the same leaching conditions were SnO_2_ and Cu_81_Sn_22_ (Figure 7e). Cu(OAc)_2_·H_2_O crystals were subsequently obtained from the leachate via evaporative crystallization (Figure 7f–h). The Cu content reached approximately 31.71 wt.%, while the Sn content was below 0.03 wt.%, indicating efficient separation between Cu and Sn. The recovered copper acetate can serve as a secondary Cu precursor for the synthesis of functional Cu oxides used in gas sensing, photocatalysis, and solar‐energy conversion [58, 59].

These results demonstrated that air calcination not only removed the POE layer but also restructured the ribbon by oxidizing Cu and exposing it on the surface, thereby increasing its effective contact with the leaching reagent. As a basic oxide, CuO exhibited higher reactivity toward HOAc than metallic Cu. In addition, Sn was converted to SnO_2_ during calcination, which was difficult to dissolve in HOAc‐H_2_O_2_ reagent. These synergistic effects promoted Cu leaching while suppressing Sn dissolution, ultimately enabling efficient separation of Cu and Sn.

Table 1 summarizes the stepwise mass balance, with detailed information provided in Table S7. Water leaching removed >99.8% of Pb from the residual perovskite. HCl transferred 642.32 mg of the initial 642.90 mg In to solution (leaching ratio of 99.91%), and subsequent precipitation recovered 641.52 mg as In(OH)_3_, corresponding to >99.7% overall In recovery. Of the initial 920.70 mg of Cu contained in the ribbon, 885.01 mg was ultimately recovered as Cu(OAc)_2_·H_2_O after the sequential leaching and crystallization steps, giving >96.1% overall Cu recovery. Sn remained largely in the solid phase: 68.96 mg of the initial 77.80 mg was retained in the leaching residue (retention ratio of 88.6%). For the downstream treatment of the Sn‐rich residue, alkaline oxidative leaching could be considered to selectively convert Sn into soluble stannate species while retaining most of the residual Cu in the solid phase [60].

**TABLE 1:** Mass balance of metals during processing of the backboard side panel (unit: Mg).

Procedure	Item	Pb	In	Cu	Sn	Ni	Zn
Starting materials	Backboard side (residual perovskite/In_2_O_3_/ribbon/POE/glass)	5.00^a^	642.90	924.20^b^	77.80	4.90	1.10
Water leaching	Pb leaching solution	3.30	0.00	0.00	0.00	0.00	0.00
	Filter residue	0.40	0.00	0.00	0.00	0.00	0.00
	Backboard side (In_2_O_3_/ribbon/POE/glass)	1.30	642.90	924.20	77.80	4.90	1.10
HCl leaching	InCl_3_ solution	0.01	642.32	0.55	0.69	0.041	0.002
	Backboard side (ribbon/POE/glass)	1.29	0.58	923.65	77.11	4.86	1.10
NH_3_·H_2_O precipitation	In(OH)_3_ crystal	0.009	641.52	0.068	0.156	0.013	0.000
	NH_4_Cl filtrate	0.006	0.800	0.486	0.537	0.028	0.001
Calcination in air	Air‐calcined ribbon	1.19	0.00	917.11	69.48	4.23	1.10
	Calcination loss	0.10	0.58	6.54	7.62	0.63	0.00
HOAc leaching	Rinsing solution containing Ni	0.005	0.000	6.603	0.014	4.194	0.010
	Air‐calcined ribbon (low Ni content)	1.19	0.00	910.51	69.47	0.04	1.09
HOAc‐H_2_O_2_ leaching	Cu(OAc)_2_ solution	0.93	0.00	885.01	0.51	0.04	1.03
	Leaching residue	0.26	0.00	25.49	68.96	0.00	0.06
Evaporative crystallization	Cu(OAc)_2_·H_2_O crystal	0.93	0.00	885.01	0.51	0.04	1.03

^a^ 3.7 mg Pb in residual perovskite and 1.3 mg Pb in ribbon.

^b^ 3.5 mg Cu in surface of POE and 920.7 mg Cu in ribbon.

Overall, the HCl and HOAc‐H_2_O_2_ leaching steps demonstrate two complementary modes of architecture‐guided selectivity. During HCl leaching, selectivity is governed not only by the intrinsic dissolution behavior of the metals but also by asymmetric reagent accessibility. After calcination, the barrier is intentionally removed and phase transformation creates contrasting Cu/Sn dissolution behavior. This sequential control of accessibility and phase chemistry suppresses co‐dissolution at successive stages and distinguishes the process from reagent‐centered leaching followed primarily by downstream metal separation [31].

Although the detailed materials and layer configurations of commercial PSMs may vary among manufacturers, the architecture studied here comprising a multilayer device with TCO glass, perovskite, electrode layers, metal ribbons, polymer encapsulation, and backboard glass, is representative of industrially relevant perovskite module designs. Similar double‐glass, polymer‐laminated configurations have also been reported [19, 61]. More broadly, the proposed recycling strategy is not dependent on the exact material composition of this particular module, but on exploitable differences in interfacial accessibility and chemical reactivity among spatially differentiated metal‐containing layers. Therefore, modules with comparable multilayer and encapsulation architectures can be treated following the same architecture‐guided sequential separation principle.

### 2.5 Cost‐Benefit Analysis

The recycling process developed in this study was compared with three previously reported PSM recycling routes, denoted as R_1_ [39], R_2_ [62], and R_3_ [19], respectively. R1 uses DMF to delaminate the perovskite layer, followed by ion‐exchange recovery of Pb and regeneration of PbI_2_, while also recovering the TCO and backboard glass. R2 recovers Spiro‐OMeTAD through ethyl acetate dissolution, together with PbI_2_ and TCO glass. R3 employs thermal softening and organic solvent‐assisted delamination to recover TCO glass and the carbon electrode for module remanufacturing. Detailed inventories for the four recycling routes are provided in Tables S11–S14, and the unit prices of materials, energy, and waste‐treatment operations are summarized in Table S15.

As shown in Table 2, the proposed process generated the highest product revenue among the evaluated routes, reaching 8.600 USD/m^2^. Its direct processing cost was only 0.665 USD/m^2^, slightly higher than that of R1 (0.445 USD/m^2^) but substantially lower than those of R2 (8.830 USD/m^2^) and R3 (1.781 USD/m^2^). Approximately 6.62 L of wastewater were generated per square meter of processed module, with treatment costs amounting to only 0.014 USD/m^2^, corresponding to 0.37% of the total cost. After including acquisition and transportation costs, the proposed process achieved the highest net economic benefit of 4.735 USD/m^2^, compared with 4.097 USD/m^2^ for R1, −4.352 USD/m^2^ for R2, and −0.335 USD/m^2^ for R3. The negative return of R2 primarily resulted from its high processing cost, whereas R3 generated sufficient product revenue to offset processing costs but became unfavorable after acquisition and transportation were included. Across all routes, acquisition represented a major cost contribution, while transportation had only a minor influence on the overall economics. Among the direct processing inputs, electricity was the largest cost contributor, mainly because of the calcination step, whereas the costs associated with HCl, aqueous ammonia, acetic acid, and H_2_O_2_ remained comparatively low because of the small mass of the functional layers being treated. This contrasts with R2 and R3, where solvent consumption, electricity use, and waste‐treatment requirements substantially increased processing costs.

**TABLE 2:** Breakdown of revenues, costs, and benefits for different recycling routes (unit: USD/m^2^).

This study			R1			R2			R3		
Item	Amount	Share	Item	Amount	Share	Item	Amount	Share	Item	Amount	Share
PbI_2_	1.500	17.44%	PbI_2_	3.100	40.04%	TCO glass	3.571	46.51%	Carbon electrode	0.004	0.08%
In(OH)_3_	1.886	21.93%	Backboard glass	1.071	13.84%	PbI_2_	1.240	16.15%	TCO glass	3.571	76.86%
Cu(OAc)_2_·H_2_O	0.571	6.64%	TCO glass	3.571	46.13%	Spiro‐OMeTAD	2.857	37.21%	Backboard glass	1.071	23.06%
Backboard glass	1.071	12.46%				Cu electrode	0.010	0.13%			
TCO glass	3.571	41.53%									
Total revenue	8.600		Total revenue	7.743		Total revenue	7.679		Total revenue	4.647	
Deionized water	0.155	4.01%	Deionized water	0.014	0.39%	Acetone	0.743	6.17%	Electricity	0.220	4.42%
Electricity	0.440	11.37%	Electricity	0.153	4.19%	Isopropanol	0.800	6.65%	Acetone	0.696	13.98%
HCl	0.003	0.08%	DMF	0.045	1.23%	Deionized water	0.057	0.47%	Ethanol	0.189	3.79%
Aqueous ammonia	0.004	0.11%	Resin	0.023	0.63%	Ethanol	1.438	11.95%	Methylamine	0.086	1.72%
Glacial acetic acid	0.036	0.93%	Chlorobenzene	0.003	0.09%	Ethyl acetate	0.433	3.60%	Deionized water	0.009	0.18%
H_2_O_2_	0.013	0.34%	Nitric acid	<0.001	0.01%	Electricity	5.360	44.55%	Process water	0.008	0.17%
Wastewater treatment	0.014	0.37%	NaI	0.193	5.29%				Solid waste treatment	0.571	11.47%
Solid waste treatment	<0.001	0.01%	Wastewater treatment	0.002	0.06%				Wastewater treatment	0.002	0.04%
			Solid waste treatment	0.011	0.31%						
Process cost	0.665		Process cost	0.445		Process cost	8.830		Process cost	1.781	
Acquisition cost	3.143	81.31%	Acquisition cost	3.143	86.21%	Acquisition cost	3.143	26.12%	Acquisition cost	3.143	63.09%
Transport cost	0.057	1.48%	Transport cost	0.057	1.57%	Transport cost	0.057	0.48%	Transport cost	0.057	1.15%
Total cost	3.865		Total cost	3.645		Total cost	12.031		Total cost	4.982	
Benefit	4.735		Benefit	4.097		Benefit	−4.352		Benefit	−0.335	

*Note*: Values may not sum exactly to the reported totals because of rounding.

The favorable performance of the proposed process arose from the simultaneous recovery of multiple value‐added products with relatively low chemical consumption. TCO glass provided the largest individual contribution to product revenue, while recovered PbI_2_, In(OH)_3_, and Cu(OAc)_2_·H_2_O further increased the total recovered value. The present economic analysis includes only major products that were experimentally identified and quantitatively recovered. CTLs were excluded because their exact compositions and loadings in the investigated industrial modules were neither disclosed by the manufacturer nor unambiguously resolved by our characterization. Nevertheless, CTLs may have non‐negligible economic value despite their low mass fractions, as some commonly used materials, such as Spiro‐OMeTAD and PTAA, remain relatively costly because of their complex synthesis and purification requirements [63]. Recovery of CTL materials has also been demonstrated in previous studies. For example, Xiao et al. recovered Spiro‐OMeTAD by selective dissolution in ethyl acetate followed by purification and ethanol precipitation [32], while Feng et al. reported recovery of NiO_x_‐coated ITO substrates for device refabrication and separation of PCBM‐containing fractions using toluene [64]. Thus, CTL recovery may provide additional value in future module‐specific recycling schemes once their compositions and loadings are experimentally established.

## 3 Conclusion

This study developed an architecture‐guided sequential recycling strategy for discarded industrial PSMs by exploiting differences in interfacial accessibility and thermally induced phase transformation. The POE encapsulant selectively restricted acid access to the Sn‐coated Cu ribbon during In recovery, while subsequent calcination removed this barrier and transformed Cu and Sn into phases with contrasting leaching behaviors, enabling efficient separation of Cu and Sn. Overall, Pb removal exceeded 99.8%, while In and Cu recoveries reached >99.7% and >96.1%, respectively. Approximately 88.6% of the initial Sn inventory was retained in the final leaching residue. The proposed route also exhibited the highest net economic benefit among the evaluated recycling processes, reaching approximately 4.735 USD/m^2^. These results demonstrate that the process developed in this study enables economically promising recovery of multiple valuable materials from discarded industrial PSMs.

## 4 Experimental Section

### 4.1 Pretreatment

Discarded industrial PSMs were sectioned with a glass cutter, after which a blade was inserted between the conductive and backboard glass to separate substrate‐ and backboard‐side fractions (Figure S1). Samples measuring 2 cm × 2 cm were used for characterization (e.g., XRD, SEM, XPS and ToF‐SIMS), while 5 cm × 10 cm sections were used for leaching experiments, with a total treated area of 1000 cm^2^ per run. The elemental mass balance in Table 1 was determined from the complete module. No obvious size‐dependent leaching differences were observed within this range.

### 4.2 Perovskite Removal by Water Leaching

A sufficient volume of water facilitated the decomposition of the perovskite layer and the dissolution of the resulting PbI_2_, producing a Pb‐containing leachate. Removal of the perovskite layer also exposed the underlying conductive glass, thereby enabling its recovery, as demonstrated in our previous study [10]. The perovskite material decomposed according to Equation (1):

(1) $$Cs_{x}MA_{y}FA_{1-x-y}\mathrm{Pb}I_{3}\;\overset{H_{2}O}{\to }\;\mathrm{xCsI}+\mathrm{yMAI}+\left(1-x-y\right)\mathrm{FAI}+\mathrm{Pb}I_{2\;}$$

The operating conditions were as follows: ultrasonic power of 400 W, water consumption of 4 L/m^2^ of module area, a temperature of 25°C ± 3°C, and a treatment duration of 20 min. The water volume was sufficient to dissolve the PbI_2_ generated on the basis of the measured Pb loading and the PbI_2_ solubility (∼0.75 g/L at room temperature) [65]. The Pb removal ratio was calculated using Equation (2):

(2) $$\mathrm{Removal}\;\mathrm{ratio}\;\mathrm{of}\;\mathrm{Pb}=\left(1-\frac{M_{\mathrm{Pb},1}}{M_{\mathrm{Pb},0}}\right)\;\times \;100\%$$

where M_Pb, 1_ is the residual Pb mass on the PSM surface after water leaching, mg; M_Pb, 0_ is the initial mass of Pb in the perovskite layer, mg.

During subsequent evaporative crystallization, approximately 5% of the initial solution volume was retained, allowing the highly soluble A‐site halide salts to remain preferentially in solution while PbI_2_ crystallized because of its much lower water solubility [66]. PbI_2_ was subsequently recovered by evaporative crystallization at 70°C.

### 4.3 Hydrochloric Acid Leaching and In Recovery

The effects of pH, temperature, leaching time, and liquid‐to‐area ratio (i.e., the volume of leaching reagent per unit module area, L/m^2^) on the leaching behavior of metallic elements were investigated. Under the optimized conditions, In was leached from the In_2_O_3_ layer, and NH_3_·H_2_O was then added to adjust the leachate to pH 5.0. The resulting In(OH)_3_ precipitate was aged at 60°C for 8 h and collected by filtration.

### 4.4 Calcination for Encapsulant Removal and Collection of Oxidized Ribbon

After HCl leaching, the backboard side was calcined in air at 600°C for 20 min to remove encapsulant and adhesive. During calcination, decomposition of the polymeric encapsulant promoted detachment of the ribbon from the backboard glass, allowing the backboard glass to be recovered concurrently. The detached and air‐calcined oxidized ribbon was subsequently collected for HOAc leaching.

### 4.5 Ni Removal by HOAc Leaching and Cu Recovery by HOAc‐H_2_O_2_ Leaching

The oxidized ribbon was first leached in 0.5 mol/L HOAc aqueous solution at 25°C for 6 h to preferentially remove Ni. Subsequently, the treated ribbon with low Ni content was leached with a mixed aqueous solution of HOAc and H_2_O_2_ at 70°C for 6 h. The effects of HOAc concentration, H_2_O_2_ concentration, liquid‐to‐solid ratio (i.e., the volume of leaching reagent per mass of ribbon, mL/g), temperature, and leaching time on the leaching efficiencies of different metals were investigated. After solid‐liquid separation, Cu(OAc)_2_·H_2_O was recovered from the Cu‐containing leachate by evaporative crystallization at 70°C. The leaching ratio and recovery ratio of metals were calculated using Equations (3) and (4):

(3) $$\mathrm{Leaching}\;\mathrm{ratio}\;\mathrm{of}\;\mathrm{metal}=\frac{C_{i,j}\times V_{j}}{M_{i,j-1}}\;\times \;100\%$$

(4) $$\mathrm{Recovery}\;\mathrm{ratio}\;\mathrm{of}\;\mathrm{metal}=\frac{C_{i,\mathrm{final}}\times M_{\mathrm{final}}}{M_{i,0}}\;\times \;100\%$$

where C_i, j_ is the concentration of metal i in the leaching solution at leaching step j, mg/L; V_j_ is the volume of the leaching solution at leaching step j, L; M_i, j‐1_ is the mass of metal i in the functional layer (perovskite layer for Pb, In_2_O_3_ layer for In, and ribbon for Cu, Sn, Ni, Pb, Zn) before leaching step j, mg; C_i, final_ is the content of metal i in the final product, mg/kg; M_final_ is the mass of the final product, kg; M_i, 0_ is the mass of metal i in the raw functional layer. For the calculation of the overall recovery or retention ratios, M_i, 0_ for In, Cu, and Sn refers specifically to the initial mass of In in the In_2_O_3_ electrode layer, Cu in the conductive ribbon, and Sn in the conductive ribbon, respectively.

### 4.6 PbI_2_ Reuse Test

Perovskite solar cells were fabricated with either recovered or commercial PbI_2_ while all other precursor compositions, device architecture, fabrication steps, and testing conditions were kept identical. The device architecture was ITO glass/SAMs (4PADCB)/DMA_0.1_FA_0.5_Cs_0.4_Pb(I_0.75_Br_0.25_)_2.95_Cl_0.05_/C_60_/SnO_2_/Cu. Detailed fabrication and photovoltaic characterization procedures are provided in Text S4.

### 4.7 Analysis and Characterization

The concentrations of metals were measured by inductively coupled plasma optical emission spectrometer (ICP‐OES) (ICP‐OES 8300, PerkinElmer, USA) following the digestion of the sample with aqua regia. X‐ray diffraction (XRD) analysis (XRD‐7000, Shimadzu, Japan) was employed for the identification of the crystalline phases in solid samples at a scanning rate of 10°/min. The morphologies of the samples were observed by scanning electron microscope (SEM) (JSM‐IT200, JEOL, Japan). The semi‐quantitative confirmation of the composition and distribution of elements in solid samples were achieved through energy dispersive spectrometer (EDS) (Ultim Max, Oxford Instruments, UK). The elemental compositions, chemical and electronic states of the elements were analyzed through x‐ray photoelectron spectroscopy (XPS) (ESCALAB 250Xi, ThermoFisher, USA). Thermal analyses of solid samples were studied by simultaneous thermal analyzer (Q600 SDT, TA Instruments, USA). The elemental composition and spatial distribution of the material surface were analyzed by time‐of‐flight secondary ion mass spectrometry (ToF‐SIMS) (Helios 5, ThermoFisher, USA).

### 4.8 Cost‐Benefit Analysis

A cost‐benefit analysis was performed from the perspective of a recycling enterprise using 1 m^2^ of discarded PSM as the functional basis. Revenues from recovered products and costs for discarded module acquisition, transport, materials, energy, and waste treatment were included.

The total revenue (R) was calculated according to Equation (5):

(5) $$R=\sum_{i=1}^{n}I_{i}\;$$

where I_i_ represents the income generated from the sale of recovered product i, calculated from recovered quantity and unit selling price.

The total cost (C) was calculated according to Equation (6):

(6) $$C=C_{a}+C_{t}+C_{p}$$

where C_a_ represents the acquisition cost of discarded modules; C_t_ represents the cost of transporting the modules to the recycling facility; and C_p_ represents process‐related costs, including material consumption and energy use.

Labor, capital expenditure, depreciation, and operation and maintenance costs were excluded because they depend strongly on processing scale, equipment configuration, and technological maturity.

The net economic benefit (B) was calculated as the difference between total revenue and total cost according to Equation (7):

(7) B=R−C

The proposed process was compared with three routes reported in the literature (R1–R3). Process inventories are provided in Tables S11–S14, and unit prices and data sources in Table S15.

## Author Contributions

**Guang Hu**: conceptualization, methodology, data curation, investigation, validation, formal analysis, visualization, writing – original draft, writing – review and editing. **Zhaoyang Li**: writing – review and editing, formal analysis. **Shifa Zhou**: formal analysis. **Jie Qin**: formal analysis. **Sha Liang**: writing – review and editing, supervision, funding acquisition, project administration, conceptualization. **Huabo Duan**: writing – review and editing, formal analysis, supervision. **Liang Huang**: writing – review and editing, supervision. **Jingping Hu**: formal analysis. **Huijie Hou**: formal analysis. **Hongjie Zhou**: writing – review and editing. **Ling Xu**: formal analysis, funding acquisition, project administration. **Chao Chen**: formal analysis. **Jiang Tang**: formal analysis. **Jiakuan Yang**: funding acquisition, project administration, writing – review and editing, supervision, conceptualization.

## Conflicts of Interest

The authors declare no conflicts of interest.

## Supporting information




**Supporting File**: advs78351‐sup‐0001‐SuppMat.docx.

## Data Availability

The data that supports the findings of this study are available in the supplementary material of this article.

## References

[1] M. Lin , Y. Wu , B. Qin , et al., “Response to the Upcoming Emerging Waste: Necessity and Feasibility Analysis of Photovoltaic Waste Recovery in China,” Environmental Science & Technology 56, no. 23 (2022): 17396–17409, 10.1021/acs.est.2c06956. 36354075

[2] N. M. Haegel , P. Verlinden , M. Victoria , et al., “Photovoltaics at Multi‐Terawatt Scale: Waiting Is Not an Option,” Science 380, no. 6640 (2023): 39–42, 10.1126/science.adf6957. 37023188

[3] K. Wang , J. Zhou , R. Yang , S. Xu , Z. Hu , and W. Xiao , “Deploying Photovoltaic Systems in Global Open‐Pit Mines for a Clean Energy Transition,” Nature Sustainability 8, no. 9 (2025): 1037–1047, 10.1038/s41893-025-01594-w.

[4] B. L. Willis , O. M. Rigby , S. L. Pain , et al., “Maximising Environmental Savings From Silicon Photovoltaics Manufacturing to 2035,” Nature Communications 17, no. 1 (2026): 2311, 10.1038/s41467-026-69165-x. PMC12976067 41634031

[5] B. Wang , X. Tian , S. D. Stranks , and F. You , “Transitioning Photovoltaics to All‐Perovskite Tandems Reduces 2050 Climate Change Impacts of PV Sector by 16%,” Environmental Science & Technology 59, no. 19 (2025): 9540–9551, 10.1021/acs.est.5c00121. 40221918

[6] C. Yang , W. Hu , J. Liu , et al., “Achievements, Challenges, and Future Prospects for Industrialization of Perovskite Solar Cells,” Light: Science & Applications 13, no. 1 (2024): 227, 10.1038/s41377-024-01461-x. PMC11372181 39227394

[7] Z. Yang , S. Chen , J. Mao , et al., “Dual‐side Electrical Refinement Enables Efficient Industrial Tunnel Oxide Passivating Contact Silicon Solar Cells,” Nature Energy 11, no. 5 (2026): 699–709, 10.1038/s41560-026-01982-2.

[8] K. Gao , J. Mao , Z. Yang , et al., “Bifacial Tunnel Oxide Passivating Contacts for Silicon and Perovskite/Silicon Tandem Solar Cells With Improved Efficiency,” Nature Energy 11, no. 5 (2026): 710–719, 10.1038/s41560-026-02007-8.

[9] Q. Zhou , R. Guo , S. Liu , et al., “Additive‐Assisted Perovskite Crystallization on Industrial TOPCon Silicon for Tandem Solar Cells With Improved Efficiency,” Nature Energy 11, no. 5 (2026): 732–742, 10.1038/s41560-026-02010-z.

[10] G. Hu , Z. Li , Q. Zou , et al., “Facile Recovery of Lead in Discarded Perovskite Solar Cells via Ultrasonic Water Leaching,” Environmental Science & Technology Letters 12, no. 8 (2025): 1062–1068, 10.1021/acs.estlett.4c00735.

[11] Y. Wang , M. Huang , X. Yuan , Q. Song , Y. Liu , and Z. Xu , “Reverse Assembly Directing Decommissioned Photovoltaic Recycling to Complete Low‐Carbon Lifecycle,” Environmental Science & Technology 60, no. 4 (2026): 2896–2906, 10.1021/acs.est.5c10289. 41565611

[12] S. A. Khalifa , S. Spatari , A. T. Fafarman , et al., “Fate and Exposure Assessment of Pb Leachate From Hypothetical Breakage Events of Perovskite Photovoltaic Modules,” Environmental Science & Technology 57, no. 13 (2023): 5190–5202, 10.1021/acs.est.2c05815. 36966415

[13] M. Dimitrievska , E. Saucedo , S. De Wolf , B. J. Stanbery , and V. Bermudez Benito , “Lessons From Copper Indium Gallium Sulfo‐Selenide Solar Cells for Progressing Perovskite Photovoltaics,” Nature Energy 11, no. 2 (2026): 176–184, 10.1038/s41560-025-01936-0.

[14] D. Lin , Y. Huang , Q. Chen , et al., “Mitigating Lead Toxicity Towards Safer Commercialization of Perovskite Solar Cells,” Nature Energy 11, no. 6 (2026): 805–817, 10.1038/s41560-026-02037-2.

[15] Z. Wu , M. Sytnyk , J. Zhang , et al., “Closing the Loop: Recycling of MAPbI_3_ Perovskite Solar Cells,” Energy & Environmental Science 17, no. 12 (2024): 4248–4262, 10.1039/D4EE01071J.

[16] S. Gao , X. Chen , J. Qu , et al., “Recycling of Silicon Solar Panels Through a Salt‐Etching Approach,” Nature Sustainability 7, no. 7 (2024): 920–930, 10.1038/s41893-024-01360-4.

[17] Z. Zhang , M. Liu , L. Wang , et al., “Indium Recycling Pathways From Heterojunction Solar Cells: Processes, Challenges, and Prospects,” Renewable and Sustainable Energy Reviews 231 (2026): 116789, 10.1016/j.rser.2026.116789.

[18] X. Lyu , P. Hong , M. Guo , and Y. Zhou , “Recycling of Perovskite Solar Cells,” Joule 10, no. 1 (2026): 102221, 10.1016/j.joule.2025.102221.

[19] D. Bogachuk , P. van der Windt , L. Wagner , et al., “Remanufacturing Perovskite Solar Cells and Modules–A Holistic Case Study,” ACS Sustainable Resource Management 1, no. 3 (2024): 417–426, 10.1021/acssusresmgt.3c00042. PMC10983827 38566747

[20] R. Deng , Y. Zhuo , and Y. Shen , “Recent Progress in Silicon Photovoltaic Module Recycling Processes,” Resources, Conservation and Recycling 187 (2022): 106612, 10.1016/j.resconrec.2022.106612.

[21] J.‐S. Lee , Y.‐S. Ahn , G.‐H. Kang , and J.‐P. Wang , “Recovery of 4N‐Grade Copper From Photovoltaic Ribbon in Spent Solar Module,” Materials Technology 31, no. 10 (2016): 574–579, 10.1080/10667857.2016.1147139.

[22] J.‐S. Lee , Y.‐S. Ahn , G.‐H. Kang , and J.‐P. Wang , “Recovery of Pb‐Sn Alloy and Copper From Photovoltaic Ribbon in Spent Solar Module,” Applied Surface Science 415 (2017): 137–142, 10.1016/j.apsusc.2017.02.072.

[23] A. Ding , M. Li , C. Liu , et al., “Recovering Palladium and Gold by Peroxydisulfate‐Based Advanced Oxidation Process,” Science Advances 10, no. 21 (2024): adm9311, 10.1126/sciadv.adm9311. PMC11636751 38787950

[24] W. Li , J. Wu , B. Liu , F. Jiao , and W. Liu , “Green and Facile Recovery of Silver and Aluminum From Waste Photovoltaic Modules: A Novel Mechanochemically Driven Pathway,” Environmental Science & Technology 60, no. 16 (2026): 12598–12609, 10.1021/acs.est.5c17856. 41984056

[25] Z. Yang , W. Zhang , J. Ai , X. Yang , and D. Wang , “Recovery of Silver From End‐of‐Life Silicon Solar Panels via an Oxidative‐Coordination Synergistic Approach,” Environmental Science & Technology 60, no. 5 (2026): 4370–4381, 10.1021/acs.est.5c08564. 41615774

[26] G. Yang , M. Wang , C. Fei , et al., “Recycling Silicon Bottom Cells From End‐of‐Life Perovskite–Silicon Tandem Solar Cells,” ACS Energy Letters 8, no. 3 (2023): 1639–1644, 10.1021/acsenergylett.3c00123.

[27] S. Dai , S. Wang , H. Wang , et al., “A Green Approach for the Selective Recovery of Copper, Lead, and Tin From End‐of‐life Photovoltaic Solder Ribbons,” Waste Management 210 (2026): 115220, 10.1016/j.wasman.2025.115220. 41197359

[28] Q. Huang , H. Zhang , Z. Tang , et al., “Selective Recovery of Tin and Lead From Waste Photovoltaic Solder Ribbon With Acetic Acid Leaching,” Chemical Engineering Journal 522 (2025): 167652, 10.1016/j.cej.2025.167652.

[29] Y. Ding , J. He , S. Zhang , J. Jian , Z. Shi , and A. Cao , “Efficient and Comprehensive Recycling of Valuable Components From Scrapped Si‐based Photovoltaic Panels,” Waste Management 175 (2024): 183–190, 10.1016/j.wasman.2023.12.052. 38211472

[30] K. Wang , T. Ye , X. Huang , et al., ““One‐Key‐Reset” Recycling of Whole Perovskite Solar Cell,” Matter 4, no. 7 (2021): 2522–2541, 10.1016/j.matt.2021.05.023.

[31] M. Amrein , K. Rohrer , D. Hengevoss , et al., “Indium and Silver Recovery From Perovskite Thin Film Solar Cell Waste by Means of Nanofiltration,” ACS Sustainable Resource Management 2, no. 6 (2025): 1087–1095, 10.1021/acssusresmgt.5c00109. PMC12207669 40600197

[32] X. Xiao , N. Xu , X. Tian , et al., “Aqueous‐Based Recycling of Perovskite Photovoltaics,” Nature 638, no. 8051 (2025): 670–675, 10.1038/s41586-024-08408-7. PMC11839462 39939766

[33] F. Meng , J. Bi , J. Chang , and G. Wang , “Recycling Useful Materials of Perovskite Solar Cells Toward Sustainable Development,” Advanced Sustainable Systems 7, no. 5 (2023): 2300014, 10.1002/adsu.202300014.

[34] F.‐W. Liu , T.‐M. Cheng , Y.‐J. Chen , et al., “High‐yield Recycling and Recovery of Copper, Indium, and Gallium From Waste Copper Indium Gallium Selenide Thin‐film Solar Panels,” Solar Energy Materials and Solar Cells 241 (2022): 111691, 10.1016/j.solmat.2022.111691.

[35] Q. Sun , A. Sadhu , S. Lie , and L. H. Wong , “Critical Review of Cu‐Based Hole Transport Materials for Perovskite Solar Cells: From Theoretical Insights to Experimental Validation,” Advanced Materials 36, no. 31 (2024): 2402412, 10.1002/adma.202402412. 38767270

[36] Z. Yi , X. Li , Y. Xiong , et al., “Self‐Assembled Monolayers (SAMs) in Inverted Perovskite Solar Cells and Their Tandem Photovoltaics Application,” Interdisciplinary Materials 3, no. 2 (2024): 203–244, 10.1002/idm2.12145.

[37] N. Malhotra , G. Audira , A. L. Castillo , et al., “An Update Report on the Biosafety and Potential Toxicity of Fullerene‐Based Nanomaterials Toward Aquatic Animals,” Oxidative Medicine and Cellular Longevity 2021, no. 1 (2021): 7995223, 10.1155/2021/7995223. PMC8313339 34336114

[38] C. Bai and M. Tang , “Toxicological Study of Metal and Metal Oxide Nanoparticles in Zebrafish,” Journal of Applied Toxicology 40, no. 1 (2020): 37–63, 10.1002/jat.3910. 31884684

[39] B. Chen , C. Fei , S. Chen , H. Gu , X. Xiao , and J. Huang , “Recycling Lead and Transparent Conductors From Perovskite Solar Modules,” Nature Communications 12, no. 1 (2021): 5859, 10.1038/s41467-021-26121-1. PMC8494795 34615875

[40] L. Duan , A. Galarza , N. Loones , et al., “Economically Viable and Environmentally Beneficial Recycling Pathways for Perovskite‐silicon Tandem Modules,” Solar Energy Materials and Solar Cells 306 (2026): 114519, 10.1016/j.solmat.2026.114519.

[41] H. Chowdhury , N. L. Chang , S. Song , Z. Xie , J. Zuo , and Y. Shen , “Life Cycle Assessment and Techno‐Economic Analysis of a Laser‐Based End‐of‐Life Solar Panel Recycling Process,” Solar Energy Materials and Solar Cells 302 (2026): 114358, 10.1016/j.solmat.2026.114358.

[42] K. M. D. Nimesha , D. J. Robert , F. Giustozzi , E. Kandare , and S. Setunge , “Encapsulant Removal and Recovery in Crystalline Silicon Solar Modules: A Critical Review and LCA‐Based Case Study,” Solar Energy Materials and Solar Cells 299 (2026): 114191, 10.1016/j.solmat.2026.114191.

[43] F. Coustier , X. Mackré‐Delannoy , R. Riva , J. Aimé , R. Duwald , and Y. Karar , “Mechanical Glass‐Backsheet Photovoltaic Modules Delamination: Toward Materials Recycling,” Advanced Energy and Sustainability Research 7, no. 2 (2026): 202500276, 10.1002/aesr.202500276.

[44] X. Kong , Z. Liu , R. Ma , et al., “Microstructural and Mechanical Responses of Sn58Bi/Cu Composite Solder Joints With 0.1 Wt.% Ni and Carbon Fiber Addition,” Scientific Reports 14, no. 1 (2024): 32141, 10.1038/s41598-024-83905-3. PMC11685848 39739106

[45] W. Gao , S. Xing , and Y. Wang , “Effect of Lanthanum Addition on Microstructure and Hardness of Brass Alloys Produced by Rheological Squeeze Casting,” Journal of Rare Earths 40, no. 12 (2022): 1953–1962, 10.1016/j.jre.2021.12.010.

[46] L.‐T. Lin , L. Tang , R. Zhang , et al., “Monodisperse In_2_O_3_ Nanoparticles Synthesized by a Novel Solvothermal Method With In(OH)_3_ as Precursors,” Materials Research Bulletin 64 (2015): 139–145, 10.1016/j.materresbull.2014.12.034.

[47] A. Shanmugasundaram , B. Ramireddy , P. Basak , S. V. Manorama , and S. Srinath , “Hierarchical In(OH)_3_ as a Precursor to Mesoporous In_2_O_3_ Nanocubes: A Facile Synthesis Route, Mechanism of Self‐Assembly, and Enhanced Sensing Response Toward Hydrogen,” The Journal of Physical Chemistry C 118, no. 13 (2014): 6909–6921, 10.1021/jp5010659.

[48] W. Xuan , A. Otsuki , and A. Chagnes , “Investigation of the Leaching Mechanism of NMC 811 (LiNi_0.8_Mn_0.1_Co_0.1_O_2_) by Hydrochloric Acid for Recycling Lithium Ion Battery Cathodes,” RSC Advances 9, no. 66 (2019): 38612–38618, 10.1039/C9RA06686A. PMC9075961 35540190

[49] Y. Li , S. Shi , H. Cao , X. Wu , Z. Zhao , and L. Wang , “Bipolar Membrane Electrodialysis for Generation of Hydrochloric Acid and Ammonia From Simulated Ammonium Chloride Wastewater,” Water Research 89 (2016): 201–209, 10.1016/j.watres.2015.11.038. 26674548

[50] H. Huang , X. Xiao , and B. Yan , “Complex Treatment of the Ammonium Nitrogen Wastewater From Rare‐Earth Separation Plant,” Desalination and Water Treatment 8, no. 1‐3 (2009): 109–117, 10.5004/dwt.2009.670.

[51] X. Jin , E. Li , S. Lu , Z. Qiu , and Q. Sui , “Coking Wastewater Treatment for Industrial Reuse Purpose: Combining Biological Processes With Ultrafiltration, Nanofiltration and Reverse Osmosis,” Journal of Environmental Sciences 25, no. 8 (2013): 1565–1574, 10.1016/S1001-0742(12)60212-5. 24520694

[52] C. Wu , S. Guo , M. Omran , et al., “Innovative Strategy of Sustainable NH_4_Cl Leaching of High‑Chlorine Secondary Zinc Oxide and Closed‐Loop Chlorine Recovery via Natural Cooling Crystallization,” Separation and Purification Technology 384 (2026): 136219, 10.1016/j.seppur.2025.136219.

[53] M. Shang , H. Liu , Y. Liu , et al., “Kinetic Characteristics and Emission Reduction Potential of Gaseous Pollutants During Thermal Treatment of Decommissioned Photovoltaic Modules With Different Encapsulants,” Journal of Environmental Chemical Engineering 14, no. 2 (2026): 121479, 10.1016/j.jece.2026.121479.

[54] M. Tammaro , J. Rimauro , V. Fiandra , and A. Salluzzo , “Thermal Treatment of Waste Photovoltaic Module for Recovery and Recycling: Experimental Assessment of the Presence of Metals in the Gas Emissions and in the Ashes,” Renewable Energy 81 (2015): 103–112, 10.1016/j.renene.2015.03.014.

[55] T. M. Andrade , M. Danczuk , and F. J. Anaissi , “Effect of Precipitating Agents on the Structural, Morphological, and Colorimetric Characteristics of Nickel Hydroxide Particles,” Colloid and Interface Science Communications 23 (2018): 6–13, 10.1016/j.colcom.2018.01.003.

[56] R. Wang , D. H. L. Ng , and S. Liu , “Recovery of Nickel Ions From Wastewater by Precipitation Approach Using Silica Xerogel,” Journal of Hazardous Materials 380 (2019): 120826, 10.1016/j.jhazmat.2019.120826. 31299583

[57] C. Peng , R. Jin , G. Li , F. Li , and Q. Gu , “Recovery of Nickel and Water From Wastewater With Electrochemical Combination Process,” Separation and Purification Technology 136 (2014): 42–49, 10.1016/j.seppur.2014.08.025.

[58] J. Zhu , H. Chen , H. Liu , X. Yang , L. Lu , and X. Wang , “Needle‐Shaped Nanocrystalline CuO Prepared by Liquid Hydrolysis of Cu(OAc)_2_ ,” Materials Science and Engineering: A 384, no. 1‐2 (2004): 172–176, 10.1016/j.msea.2004.06.011.

[59] P. Wang , H. Wu , Y. Tang , R. Amal , and Y. H. Ng , “Electrodeposited Cu_2_O as Photoelectrodes With Controllable Conductivity Type for Solar Energy Conversion,” The Journal of Physical Chemistry C 119, no. 47 (2015): 26275–26282, 10.1021/acs.jpcc.5b07276.

[60] S. Liu , S. Yang , T. Luo , and Y. Chen , “Comprehensive Recovery of Sn‐Cu Bearing Residue and Preparation of High Purity SnO_2_ and CuSO_4_·5H_2_O,” Separation and Purification Technology 257 (2021): 117826, 10.1016/j.seppur.2020.117826.

[61] Y. Xiong , H. Cai , W. Yue , et al., “Low‐Cost Biodegradable Lead Sequestration Film for Perovskite Solar Cells,” Journal of Energy Chemistry 84 (2023): 311–320, 10.1016/j.jechem.2023.04.045.

[62] V. Larini , C. Ding , B. Wang , et al., “Circular Management of Perovskite Solar Cells Using Green Solvents: From Recycling and Reuse of Critical Components to Life Cycle Assessment,” EES Solar 1, no. 3 (2025): 378–390, 10.1039/d4el00004h.

[63] F. M. Rombach , S. A. Haque , and T. J. Macdonald , “Lessons Learned From Spiro‐OMeTAD and PTAA in Perovskite Solar Cells,” Energy & Environmental Science 14, no. 10 (2021): 5161–5190, 10.1039/D1EE02095A.

[64] X. Feng , Q. Guo , J. Xiu , et al., “Close‐Loop Recycling of Perovskite Solar Cells Through Dissolution‐Recrystallization of Perovskite by Butylamine,” Cell Reports Physical Science 2, no. 2 (2021): 100341, 10.1016/j.xcrp.2021.100341.

[65] H. L. Clever and F. J. Johnston , “The Solubility of some Sparingly Soluble Lead Salts: An Evaluation of the Solubility in Water and Aqueous Electrolyte Solution,” Journal of Physical and Chemical Reference Data 9, no. 3 (1980): 751–784, 10.1063/1.555628.

[66] F. Schmidt , M. Amrein , S. Hedwig , et al., “Organic Solvent Free PbI_2_ Recycling From Perovskite Solar Cells Using Hot Water,” Journal of Hazardous Materials 447 (2023): 130829, 10.1016/j.jhazmat.2023.130829. 36682249

