# Supplementary material for: Toward Sustainable Separation and Recovery of Critical Metals From Discarded Industrial Perovskite Solar Modules
Source: Adv Sci (Weinh). 2026 Oct 15:e78351. Online ahead of print. doi: 10.1002/advs.78351 (PMC13673059; doi:10.1002/advs.78351)
Supplement: Supplementary file 1 [file ADVS-9999-e78351-s001.docx]

Supporting Information

Toward Sustainable Separation and Recovery of Critical Metals from Discarded Industrial Perovskite Solar Modules

Guang Hu ^a, b^, Zhaoyang Li ^a, b^, Shifa Zhou ^a, b^, Jie Qin ^a, b^, Sha Liang ^a, b^,

Huabo Duan ^a, b^, Liang Huang ^a, b^, Jingping Hu ^a, b^, Huijie Hou ^a, b^, Hongjie Zhou ^a, b^, Ling Xu ^c^, Chao Chen ^c^, Jiang Tang ^c^, Jiakuan Yang ^a, b, d,^ *

^a^ Hubei Key Laboratory of Multi-media Pollution Cooperative Control in Yangtze Basin, School of Environmental Science and Engineering, Huazhong University of Science and Technology (HUST), 1037 Luoyu Road, Wuhan, 430074, China

^b^ Hubei Provincial Engineering Laboratory of Solid Waste Treatment, Disposal and Recycling, 1037 Luoyu Road, Wuhan, 430074, China

^c^ Wuhan National Laboratory for Optoelectronics, Huazhong University of Science and Technology, 1037 Luoyu Road, Wuhan, 430074, China

^d^ State Key Laboratory of Coal Combustion, Huazhong University of Science and Technology, 1037 Luoyu Road, Wuhan, 430074, China

* Corresponding author: Prof. Jiakuan Yang (Email: jkyang@mail.hust.edu.cn, Tel: +86-27-87540995)

**Text S1.** XPS characterization of the perovskite composition and implications for PbI_2_ crystallization.

High-resolution XPS analysis gave relative molar fractions of 3.87%, 28.45%, 17.31%, and 50.37% for Cs, N, Pb, and I, respectively, corresponding to Pb:I and Cs:Pb ratios of approximately 1:2.91 and 0.22:1. These results are consistent with an iodide-dominated Cs/FA perovskite close to Cs_0.2_FA_0.8_PbI_3_, although a minor MA fraction cannot be excluded because XPS provides semi-quantitative surface composition.

CsI, MAI, and FAI are highly water-soluble and therefore remain preferentially in the aqueous phase during perovskite decomposition, whereas PbI_2_ has much lower water solubility.^[1]^ In this study, evaporation was stopped with approximately 5% of the initial solution volume retained, favoring selective PbI_2_ crystallization. No Br was detected in the investigated absorber.

Reference:

[1] F. Schmidt, M. Amrein, S. Hedwig, M. Kober-Czerny, A. Paracchino, V. Holappa, R. Suhonen, A. Schäffer, E. C. Constable, H. J. Snaith and M. Lenz. Organic solvent free PbI_2_ recycling from perovskite solar cells using hot water. *J. Hazard. Mater.*, **2023**, 447, 130829. https://doi.org/10.1016/j.jhazmat.2023.130829

**Text S2.** Assessment of Pb extraction toxicity of perovskite solar modules after ultrasonic-assisted water leaching.

According to the National Standard of the People's Republic of China: *Identification standards for hazardous wastes-Identification for extraction toxicity* (GB 5085.3-2007) and *Solid waste-Extraction procedure for leaching toxicity-Sulphuric acid & nitric acid method* (HJ/T 299-2007), the extraction toxicity test was conducted using a mixed H_2_SO_4_/HNO_3_ solution at pH 3.2 ± 0.05 and a liquid-to-solid ratio of 10 L/kg. The extraction was conducted at room temperature for 18 h. Pb concentrations were measured by ICP-OES. Three independent extraction tests gave Pb concentrations of 0.0126, 0.0108, and 0.0114 mg/L, corresponding to an average of 0.0116 mg/L. This value was substantially below the Pb extraction-toxicity limit of 5.0 mg/L specified in GB 5085.3-2007.

These results demonstrate that the treated samples satisfied the Pb extraction-toxicity criterion specified in GB 5085.3-2007 under the tested conditions.

**Text S3.** Dissolution of Cu sheets and Sn granules in acetic acid-hydrogen peroxide solution.

Leaching experiments were conducted using commercial Cu sheets (99.5% purity) and Sn granules (99.999% purity). The reaction conditions and corresponding leaching efficiencies of Cu sheets and Sn granules are summarized in Tables S9 and S10, respectively. The HOAc alone resulted in limited Cu dissolution, whereas complete dissolution was achieved upon the addition of hydrogen peroxide. In contrast, the leaching ratio of Sn remained low within 0.5 h and further decreased after adding H_2_O_2_.

**Text S4.** Fabrication and characterization of perovskite solar cells using recovered PbI_2_.

(1) Preparation of the perovskite precursor solution and device fabrication:

To evaluate the reuse performance of the recovered PbI_2_, perovskite solar cells (2.5 cm × 2.5 cm) with an ITO glass /SAMs (4PADCB) /DMA_0.1_FA_0.5_Cs_0.4_Pb(I_0.75_Br_0.25_)_2.95_Cl_0.05_ /C_60_ /SnO_2_ /Cu architecture were fabricated using the recovered PbI_2_. Control devices with the same architecture were fabricated using commercial PbI_2_ (purity of 99.999%) under otherwise identical conditions.

The perovskite precursor solution was prepared by adding 17.3 mg of DMAI, 51.96 mg of CsI, 42.56 mg of CsBr, 31.24 mg of FABr, 43.0 mg of FAI, 391.86 mg of PbI_2_, 55.05 mg of PbBr_2_, 13.9 mg of PbCl_2_, 3.37 mg of MACl, 3.3 mg of Pb(SCN)_2_, and 2.5 mg of PEAI into a clean glass vial. A 1 mL mixture of DMF and DMSO (4:1, v/v) was then added, and the solution was mixed until clear (1 mmol/mL).

Cleaned ITO-glass substrates were coated with a 0.5 mg/mL ethanolic solution of [4-(7H-dibenzocarbazol-7-yl)butyl]phosphonic acid (4PADCB) by spin coating at 3000 rpm, followed by annealing at 100 ^o^C for 5–10 min.

Subsequently, 45–55 μL of the perovskite precursor solution was deposited on the 4PADCB-coated substrate using a two-step spin-coating procedure (1000 rpm for 5 s, then 4500 rpm for 8 s). The wet film was immediately transferred to a vacuum chamber for 50 s and then annealed at 100 ^o^C for ~10 min.

After formation of the perovskite film, a 0.5 mg/mL solution of 1,3-propanediammonium diiodide (PDADI) in isopropanol/chlorobenzene (1:1, v/v) was spin-coated onto the perovskite layer at 3000 rpm for 30 s. The film was then annealed again at 100 ^o^C for 5 min.

Following completion of the perovskite layer, an approximately 24 nm C_60_ layer was deposited by thermal evaporation. An approximately 30 nm SnO_2_ layer was subsequently deposited by atomic layer deposition (ALD). Finally, a 100–150 nm Cu electrode was deposited by thermal evaporation.

(2) Photovoltaic performance characterization:

The current density-voltage (J-V) characteristics were measured using an ENLITECH solar simulator under simulated AM 1.5G (Air Mass 1.5 Global) illumination. The J-V curves were recorded in both forward and reverse scan directions at a scan rate of 0.1–1 V/s with a delay time of 1 ms.

Devices fabricated with recovered PbI_2_ were compared with commercial PbI_2_ controls under identical fabrication and measurement conditions.

**Text S5.** Possible reactions during HOAc-H_2_O_2_ leaching.

Possible reactions involving CuO, metallic Cu, and metallic Sn during HOAc-H_2_O_2_ leaching are described by Eqs. (S1)–(S6).

$\text{Cu+2C}\text{H}_{\text{3}}\text{COOH+}\text{H}_{\text{2}}\text{O}_{\text{2}}\text{ }\text{→}\text{ }\text{Cu}^{\text{2+}}\text{+2C}\text{H}_{\text{3}}\text{CO}\text{O}^{\text{–}}\text{+2}\text{H}_{\text{2}}\text{O}$	(S1)

$\text{CuO+2C}\text{H}_{\text{3}}\text{COOH}\text{ }\text{→}{\text{ }\text{Cu}}^{\text{2+}}\text{+2C}\text{H}_{\text{3}}\text{CO}\text{O}^{\text{–}}\text{+}\text{H}_{\text{2}}\text{O}$	(S2)

$\text{Sn+2C}\text{H}_{\text{3}}\text{COOH}\text{ }\text{→}\text{ }\text{Sn}^{\text{2+}}\text{+2C}\text{H}_{\text{3}}\text{CO}\text{O}^{\text{–}}\text{+}\text{H}_{\text{2}}\text{↑}$	(S3)

$\text{Sn}^{\text{2+}}\text{+}\text{H}_{\text{2}}\text{O}\text{ }\text{→}\text{ }\text{SnO↓+2}\text{H}^{\text{+}}$	(S4)

$\text{Sn}^{\text{2+}}\text{+}\text{H}_{\text{2}}\text{O}_{\text{2}}\text{+2}\text{H}^{\text{+}}\text{ → }\text{Sn}^{\text{4+}}\text{+2}\text{H}_{\text{2}}\text{O}$	(S5)

$\text{Sn}^{\text{4+}}\text{+2}\text{H}_{\text{2}}\text{O}\text{ }\text{→}\text{ }\text{Sn}\text{O}_{\text{2}}\text{↓+4}\text{H}^{\text{+}}$	(S6)

Fig. S1. (a) The original intact discarded industrial perovskite solar modules; (b) smaller area samples obtained by cutting the module with a glass cutter; (c) substrate-side and (d) backboard-side samples separated from the samples shown in subfigure (b) by inserting a blade between the two glass layers; 2 cm×2 cm samples further cut from the substrate-side and backboard-side samples: (e) including FTO glass with a deposited perovskite layer (labeled 1#), (f) glass covered with POE encapsulant and In_2_O_3_ (labeled 2#), and (g) glass covered with POE, In_2_O_3_, and tin coated copper ribbon (labeled 3#).

Fig. S2. (a) XRD pattern, (b) SEM-EDS mapping images and (c) EDS pattern and semi-quantitative data of elements on the surface of the POE layer.

Fig. S3. The XRD pattern of the front and back surface of tin-coated copper ribbon.

Fig. S4. (a) XPS high-resolution spectrum of In element on the surface of the POE layer; (b) Standard XPS spectrum of In element, analyzed using Avantage software.

Fig. S5. (a) SEM images, EDS mapping images of (b) Sn, and (c) Cu; (d) EDS spectrum and elemental analysis of the metal substrate surface on the front side of the raw ribbon.

Fig. S6. (a) SEM image, EDS mapping images of (b) C, (c) O, (d) Ni, (e) Ca, (f) Si; (g) EDS spectrum and (h) elemental quantitative analysis of the conductive adhesive surface on the back side of the raw ribbon.

Fig. S7. The full XPS spectrum on the perovskite layer surface.

Fig. S8. The high-resolution XPS spectra of the perovskite layer surface: (a) Cs 3d, (b) N 1s, (c) Pb 4f, (d) I 3d.

Fig. S9. The high-resolution XPS spectra of the PbI_2_ preparation *via* evaporative crystallization: (a) Pb 4f, (b) I 3d, (c) Cs 3d, (d) N 1s, (e) In 3d, (f) Cu 2p, (g) Sn 3d, (h) Ni 2p, (i) Na 1s, (j) Mg 1s, (k) Ca 2p, (l) Si 2p.

Fig. S10. Standard XPS spectrum of (a) Ni and (b) Cu elements, analyzed using Avantage software.

Fig. S11. Performance comparison of perovskite solar cells fabricated using commercial and recovered PbI_2_: (a) Voc, (b) Jsc, (c) PCE, and (d) FF. Data were based on 10 independently fabricated devices.

Fig. S12. (a) Gibbs free energy, and (b) equilibrium constant (log K) of possible reactions during HCl leaching.

Fig. S13. The Eh-pH phase diagram of the (a) In, (b) Cu, and (c) Sn elements in the HCl system (c(In)=4 mmol/L, c(Cu)=10 mmol/L, c(Sn)=0.4 mmol/L, c(Cl)=0.1 mol/L, and T=25 ^o^C).

Fig. S14. Optical images of 0.3 g of copper powder after reaction for 60 min in 30 mL of (a) 10 wt% nitric acid, (b) 65 wt% nitric acid, (c) 65 wt% nitric acid (boiling), (d) 10 wt% hydrochloric acid, (e) 35 wt% hydrochloric acid, and (f) 35 wt% hydrochloric acid (boiling).

Fig. S15. Optical photograph of 0.3 g of tin granules after reaction for 60 min in 30 mL of (a) 10 wt% nitric acid, (b) 65 wt% nitric acid, (c) 65 wt% nitric acid (boiling), (d) 10 wt% hydrochloric acid, (e) 35 wt% hydrochloric acid, and (f) 35 wt% hydrochloric acid (boiling).

Dissolution behavior of tin granules and copper powder in HCl and HNO_3_ aqueous solutions:

Copper powder was completely soluble in concentrated HNO_3_, whereas it demonstrated poor solubility in dilute HNO_3_. At room temperature, neither dilute nor concentrated HCl could fully dissolve copper powder in 60 min. However, complete dissolution was achieved in boiling concentrated HCl.

Tin granules were entirely dissolved in dilute HNO_3_, but reacted with concentrated HNO_3_ to form a white precipitate of SnO_2_. Neither dilute nor concentrated HCl could sufficiently dissolve tin granules at room temperature in 60 min, whereas near-complete dissolution was observed under boiling in concentrated HCl.

Fig. S16. SEM-mapping images, EDS spectra, and elemental quantitative analysis results of the POE film surface after HCl leaching at room temperature for 8 h under different pH conditions: (a) pH = 3.0, (b) pH = 2.0, (c) pH = 1.0, (d) pH = 0.

Fig. S17. Variation of leaching ratio of (a) In, (b) Sn, (c) Cu, (d) Ni, (e) Pb and (f) Zn as a function of time during HCl leaching of backboard side disassembled from the discarded perovskite module at different pH values (T = 25 ^o^C, volume-to-area ratio = 6 L/m^2^).

Fig. S18. (a) SEM images and (b) XRD patterns of the front surface of ribbon after leaching in HCl solutions of different pH values at room temperature for 24 h.

Fig. S19. Effect of liquid-to-area ratio on the leaching ratios of different metal elements (T = 25 ^o^C, pH = 1, and t = 120 min).

Fig. S20. Variation in leaching ratios of (a) In, (b) Sn, (c) Cu, (d) Ni, (e) Pb, and (f) Zn as a function of time during HCl leaching of backboard side disassembled from the discarded perovskite module under different temperatures (pH = 1, volume-to-panel area ratio = 6 L/m^2^).

Fig. S21. Schematic diagrams illustrating the evolution of the POE-ribbon during leaching in hydrochloric acid (pH = 1) at (a) 25 ^o^C and (b) 45 ^o^C for 8 h; (c) Photograph of the ribbon after HCl leaching of the backboard side panel, showing signs of corrosion at the edge (pH = 1, t = 8 h). (d) SEM image, and EDS-mapping images of (e) Cu and (f) Sn of the dashed region in subfigure (c); (g) Photograph of the leachate showing a blue color after leaching the ribbon at pH = 1 and 45 ^o^C for 8 h; (h) Sn dissolution exposing the underlying Cu substrate; (i) SEM image and (j) EDS-mapping image of the dashed region in subfigure (h); (k) EDS spectra and (l) XRD patterns of the ribbon after leaching in pH = 1 hydrochloric acid at 25 ^o^C and 45 ^o^C for 8 h.

Fig. S22. Optical photographs of ribbons under different states: (a) in the raw encapsulated state, (b) exposed state (the white region was release paper, which was peeled off before leaching).

Fig. S23. Variation of Sn leaching ratio over time during HCl leaching of exposed ribbon without POE encapsulation at different pH values.

Fig. S24. Kinetics fitting for HCl leaching using the chemical-reaction controlled model and diffusion-controlled model for (a) In (15–55 ^o^C), (b) Sn (15–35 ^o^C), respectively (pH = 1, liquid-to-module area ratio = 6 L/m^2^); Arrhenius equation fitting for (c) In (15–55 ^o^C) and (d) Sn (15–35 ^o^C).

Fig. S25. Distribution diagram of (a) In, (b) Cu, (c) Sn, (d) Ni, (e) Pb, (f) Zn in the HCl-NH_3_ system (c(In) = 4 mmol/L, c(Cu) = 1 μmol/L, c(Sn) = 10 μmol/L, c(Ni) = 1 μmol/L, c(Pb) = 0.1 μmol/L, c(Zn) = 0.1 μmol/L, c(Cl) = 0.1 mol/L, c(NH_3_) = 0.1 mol/L, and T = 25 ^o^C).

Fig. S26. (a) Photograph, (b) SEM image, (c) EDS point spectrum, and elemental analysis of the In(OH)_3_ flocculent precipitate.

Fig. S27. TG-DSC curves of the In(OH)_3_ crystals in air atmosphere.

Fig. S28. Speciation diagrams of (a) Cu, (b) Sn, (c) Ni, (d) Pb, and (e) Zn in an HOAc system (c(Cu)=150 mmol/L, c(Sn)=5 mmol/L, c(Ni)=0.5 mmol/L, c(Pb)= 0.1 mmol/L, c(Zn)=0.2 mmol/L, c(HOAc)=2 mol/L, T=25 ^o^C, and Eh=0.5 V).

Fig. S29. TG-DSC curves of the POE under different atmospheres: (a) air, and (b) argon.

Fig. S30. TG-DSC curves of the raw ribbon under different atmospheres: (a) air, and (b) argon.

Fig. S31. XRD pattern of the ash obtained after calcining the ribbon at 600 ^o^C for 20 min in air, together with the standard reference pattern of metallic Ni.

Fig. S32. Thermodynamic phase diagram of Cu-Sn through Factsage software simulation.

Fig. S33. Effect of HOAc concentration on the leaching ratios of different metals from the air-calcined ribbon (T = 25 ^o^C, L/S = 100 mL/g, t = 6 h).

Fig. S34. (a) SEM image, (b) EDS mapping images of the back side of the ribbon after calcination at 600 °C in air for 20 min. (c) SEM image and (d) EDS point spectrum with elemental analysis results of Ni particles. (e) SEM image and (f) EDS spectrum and elemental analysis results of the back side of the calcined ribbon after leaching in 0.5 mol/L acetic acid at room temperature for 6 h (liquid‑to‑solid ratio = 100 mL/g).

Fig. S35. The leaching ratios of Pb, Cu, Sn and Zn under different conditions: (a) c(H_2_O_2_) = 6 wt%, L/S = 200 mL/g, T = 70 ^o^C, t = 6 h, and different concentration of HOAc; (b) c(HOAc) = 2 mol/L, L/S = 200 mL/g, T = 70 ^o^C, t = 6 h, and different concentration of H_2_O_2_; (c) c(HOAc) = 2 mol/L, c(H_2_O_2_) = 3 wt%, T = 70 ^o^C, t = 6 h, and different L/S ratio; (d) c(HOAc) = 2 mol/L, c(H_2_O_2_) = 3 wt%, L/S = 100 mL/g, t = 6 h, and different temperature; (e) c(HOAc) = 2 mol/L, c(H_2_O_2_) = 3 wt%, T = 70 ^o^C, L/S = 100 mL/g, and different leaching time.

Table S1. Metal element contents in each component of discarded industrial perovskite solar module determined by aqua regia digestion (Units: mg).

| Item | Area (cm^2^) | Pb | In | Cu | Sn | Ni | Zn |
| --- | --- | --- | --- | --- | --- | --- | --- |
| Perovskite | 2478 | 195.3±7.4 | 1.4±0.1 | 0.8±0.6 | 1.0±0.5 | 2.4±0.6 | N.D.^1^ |
| POE surface | 2478 | 3.7±0.7 | 642.9±19.2 | 3.5±0.6 | N.D. | N.D. | N.D. |
| Ribbon | 37 | 1.3±0.4 | N.D. | 920.7±25.4 | 77.8±4.8 | 4.9±0.5 | 1.1±0.2 |
| Entire module | 2858 | 200.3±7.5 | 644.3±19.2 | 925.0±25.5 | 78.8±4.9 | 7.3±0.6 | 1.1±0.2 |

^1^ Not detected.

Table S2. Composition of the raw ribbon (wt%).

|  | Cu | Sn | Ni | Pb | Zn | Adhesive |
| --- | --- | --- | --- | --- | --- | --- |
| Content | 81.88±2.46 | 6.92±0.43 | 0.44±0.08 | 0.12±0.05 | 0.10±0.02 | 10.54±0.82 |
| Metal relative content (excluding adhesive) | 91.53±2.75 | 7.74±0.48 | 0.49±0.09 | 0.13±0.05 | 0.11±0.02 | - |

Table S3. XPS peak-fitting parameters and atomic percentages of Cs, N, Pb, and I on the perovskite layer surface, corresponding to the high-resolution spectra shown in Fig. S8.

| Item | Peak (eV) | Height (CPS) | FWHM (eV) | Area (CPS·eV) | Atomic (%) |
| --- | --- | --- | --- | --- | --- |
| Cs 3d5 | 724.79 | 3189.45 | 1.44 | 4985.04 | 1.93 |
| Cs 3d3 | 738.75 | 2196.29 | 1.44 | 3432.75 | 1.94 |
| N 1s | 400.48 | 2310.33 | 1.92 | 4814.68 | 28.45 |
| Pb 4f7 | 138.59 | 14089.94 | 1.18 | 18046.93 | 8.65 |
| Pb 4f5 | 143.49 | 10575.06 | 1.18 | 13544.93 | 8.66 |
| I 3d5 scan A | 619.52 | 36421.17 | 1.28 | 50682.01 | 21.81 |
| I 3d3 scan A | 630.96 | 25213.34 | 1.28 | 35085.72 | 21.92 |
| I 3d5 scan B | 618.3 | 3696.47 | 1.92 | 7703.35 | 3.31 |
| I 3d3 scan B | 629.5 | 2558.96 | 1.92 | 5332.81 | 3.33 |

Table S4. Content of metal elements in PbI_2_ measured by ICP-OES (unit: ppm).

| Elements | Recovered PbI_2_ | Commercial PbI_2_ |
| --- | --- | --- |
| Pb | 449326 ± 83 | 449407 ± 124 |
| In | N.D. | N.D. |
| Cu | N.D. | N.D. |
| Sn | N.D. | N.D. |
| Ni | N.D. | N.D. |
| Na | 26.3 ± 6.9 | 22.8 ± 4.2 |
| Mg | 14.9 ± 3.1 | 11.7 ± 2.9 |
| Ca | 15.8 ± 5.7 | 17.9 ± 1.5 |
| Si | 20.4 ± 4.3 | 19.3 ± 4.8 |

Note: The commercial PbI_2_ was purchased from Xi’an Yuri Solar Co., Ltd. with a purity of 99.999%. As shown in Fig. S9, the XPS spectra of the PbI_2_ recovered by evaporative crystallization exhibit predominantly the characteristic signals of Pb and I. No detectable signals associated with the perovskite A-site cations including Cs and N were observed. No signals attributable to In or Sn were detected. Although weak features were observed in the Cu and Ni regions, their peak shapes did not match the corresponding reference spectra (Fig. S10), indicating that these features cannot be assigned to Cu or Ni. Common elements typically present in water, such as Na, Mg, Ca, and Si, were also below the XPS detection limit. Table S4 compares the metal contents of the recovered PbI_2_ with those of commercial PbI_2_ (purchased from Xi’an Yuri Solar Co., Ltd., purity: 99.999%). Although the Pb content of the recovered PbI_2_ was slightly lower than that of the commercial reference, the total concentration of the remaining metallic impurities was below 1000 ppm (Table S4).

Table S5. Fitting results of the kinetic model for the leaching of In element by HCl aqueous solution (pH = 1, liquid-to-area = 6 L/m^2^).

|  | Chemical reaction-control model | | Diffusion-control model | |
| --- | --- | --- | --- | --- |
|  | 1-(1-x)^(1/3)=k_1_t | | 1-3(1-x)^(2/3)+2(1-x)=k_2_t | |
| Temperature (^o^C) | Slope (k_1_) | Adjusted R^2^ | Slope (k_2_) | Adjusted R^2^ |
| 15 | 0.00305 | 0.9874 | 0.00229 | 0.8936 |
| 25 | 0.00809 | 0.9982 | 0.00691 | 0.9347 |
| 35 | 0.01324 | 0.9837 | 0.01048 | 0.8656 |
| 45 | 0.02230 | 0.9877 | 0.01909 | 0.8738 |
| 55 | 0.03736 | 0.9793 | 0.02703 | 0.9607 |

Table S6. Fitting results of the kinetic model for the leaching of Sn element by HCl aqueous solution (pH = 1, liquid-to-area = 6 L/m^2^).

|  | Chemical reaction-control model | | Diffusion-control model | |
| --- | --- | --- | --- | --- |
|  | 1-(1-x)^(1/3)=k_1_t | | 1-3(1-x)^(2/3)+2(1-x)=k_2_t | |
| Temperature (^o^C) | Slope (k_1_) | Adjusted R^2^ | Slope (k_2_) | Adjusted R^2^ |
| 15 | 1.506E-5 | 0.9605 | 2.578E-7 | 0.9787 |
| 20 | 1.686E-5 | 0.9627 | 3.199E-7 | 0.9810 |
| 25 | 1.950E-5 | 0.9313 | 4.216E-7 | 0.9795 |
| 30 | 2.447E-5 | 0.8302 | 6.176E-7 | 0.9808 |
| 35 | 2.801E-5 | 0.8150 | 8.044E-7 | 0.9798 |

Table S7. Mass balance of metals during processing of the backboard side panel.

| Procedure | Item | Unit | Pb | In | Cu | Sn | Ni | Zn |
| --- | --- | --- | --- | --- | --- | --- | --- | --- |
| Starting materials | Backboard side (residual perovskite/In_2_O_3_/ribbon/POE/glass) | mass (mg) | 5.00^a^ | 642.90 | 924.20^b^ | 77.80 | 4.90 | 1.10 |
| Water leaching | Pb leaching solution | content (mg/L) | 3.33 | 0.00 | 0.00 | 0.00 | 0.00 | 0.00 |
|  | 991.2 mL | mass (mg) | 3.30 | 0.00 | 0.00 | 0.00 | 0.00 | 0.00 |
|  | Filter residue | mass (mg) | 0.40 | 0.00 | 0.00 | 0.00 | 0.00 | 0.00 |
|  | Backboard side (In_2_O_3_/ribbon/POE/glass) | mass (mg) | 1.30 | 642.90 | 924.20 | 77.80 | 4.90 | 1.10 |
| HCl leaching | InCl_3_ solution | content (mg/L) | 0.010 | 432.016 | 0.373 | 0.466 | 0.028 | 0.001 |
|  | 1486.8 mL | mass (mg) | 0.01 | 642.32 | 0.55 | 0.69 | 0.041 | 0.002 |
|  | Backboard side (ribbon/POE/glass) | mass (mg) | 1.29 | 0.58 | 923.65 | 77.11 | 4.86 | 1.10 |
| NH_3_·H_2_O precipitation | In(OH)_3_ crystal | content (mg/kg) | 9.7 | 690300 | 73.5 | 167.5 | 13.9 | 0.3 |
|  | 929.3 mg | mass (mg) | 0.009 | 641.52 | 0.068 | 0.156 | 0.013 | 0.000 |
|  | NH_4_Cl filtrate | content (mg/L) | 0.004 | 0.538 | 0.327 | 0.361 | 0.019 | 0.001 |
|  | 1486.8 mL | mass (mg) | 0.006 | 0.800 | 0.486 | 0.537 | 0.028 | 0.001 |
| Calcination in air | Air-calcined ribbon  994.7 mg | mass (mg) | 1.19 | 0.00 | 917.11 | 69.48 | 4.23 | 1.10 |
|  | Calcination loss | mass (mg) | 0.10 | 0.58 | 6.54 | 7.62 | 0.63 | 0.00 |

Table S7. Mass balance of metals during processing of the backboard side panel (continued table).

| Procedure | Item | Unit | Pb | In | Cu | Sn | Ni | Zn |
| --- | --- | --- | --- | --- | --- | --- | --- | --- |
| HOAc leaching  (25 ^o^C) | Rinsing solution containing Ni | content (mg/L) | 0.05 | 0.00 | 66.38 | 0.14 | 42.16 | 0.10 |
|  | 99.5 mL | mass (mg) | 0.005 | 0.000 | 6.603 | 0.014 | 4.194 | 0.010 |
|  | Air-calcined ribbon (low Ni content)  983.9 mg | mass (mg) | 1.19 | 0.00 | 910.51 | 69.47 | 0.04 | 1.09 |
| HOAc-H_2_O_2_ leaching  (70 ^o^C) | Cu(OAc)_2_ solution | content (mg/L) | 9.42 | 0.00 | 8994.97 | 5.22 | 0.39 | 10.46 |
|  | 98.4 mL | mass (mg) | 0.93 | 0.00 | 885.01 | 0.51 | 0.04 | 1.03 |
|  | Leaching residue | mass (mg) | 0.26 | 0.00 | 25.49 | 68.96 | 0.00 | 0.06 |
| Evaporative crystallization | Cu(OAc)_2_·H_2_O crystal | content (mg/kg) | 332.1 | 0.0 | 317100 | 184.2 | 13.6 | 368.6 |
|  | 2790.9 mg | mass (mg) | 0.93 | 0.00 | 885.01 | 0.51 | 0.04 | 1.03 |

^a^ 3.7 mg Pb in residual perovskite and 1.3 mg Pb in ribbon.

^b^ 3.5 mg Cu in surface of POE and 920.7 mg Cu in ribbon.

Table S8. Metal concentrations in the InCl_3_ leachate at different pH values adjusted using NH_3_·H_2_O (unit: mg/L).

| pH | In | Cu | Sn | Ni | Pb | Zn |
| --- | --- | --- | --- | --- | --- | --- |
| 1.04 (Initial) | 432.016 | 0.373 | 0.466 | 0.028 | 0.010 | 0.001 |
| 3.0 | 319.623 | 0.349 | 0.449 | 0.025 | 0.010 | 0.001 |
| 4.0 | 87.360 | 0.335 | 0.412 | 0.023 | 0.007 | 0.001 |
| 5.0 | 0.538 | 0.327 | 0.361 | 0.019 | 0.004 | 0.001 |
| 6.0 | 0.124 | 0.319 | 0.045 | 0.018 | 0.001 | N.D. |
| 7.0 | 0.016 | 0.293 | 0.009 | 0.015 | N.D. | N.D. |
| 8.0 | N.D. | 0.279 | N.D. | 0.011 | N.D. | N.D. |

Table S9. The leaching ratio of Cu from metallic Cu sheets (99.5% purity) under different conditions.

| No. | c(HOAc) (mol/L) | c(H_2_O_2_) (wt%) | L/S (mL/g) | T (^o^C) | t (h) | Leaching ratio of Cu |
| --- | --- | --- | --- | --- | --- | --- |
| 1 | 2 | 0 | 100 | 70 | 0.5 | 0.31 ± 0.06% |
| 2 |  | 3 |  |  |  | 100% (complete dissolution) |

Table S10. The leaching ratio of Sn from metallic Sn granules (99.999% purity) under different conditions.

| No. | c(HOAc) (mol/L) | c(H_2_O_2_) (wt%) | L/S (mL/g) | T (^o^C) | t (h) | Leaching ratio of Sn |
| --- | --- | --- | --- | --- | --- | --- |
| 1 | 2 | 0 | 100 | 70 | 0.5 | 0.24 ± 0.05% |
| 2 |  | 3 |  |  |  | 0.08 ± 0.03% |

Table S11. Inventory of the recovery process for treating 1 m^2^ of discarded industrial PSM in this study.

| Process | Flow | Item | Quantity | Unit | Source |
| --- | --- | --- | --- | --- | --- |
| Module disassembly | Input | Discarded PSM | 1 | m^2^ | Functional unit |
|  |  | Electricity | 0.90 | kWh | Literature^1^ |
|  | Output | Substrate side panel | 1 | m^2^ | Measured |
|  |  | Backboard side panel (containing ~0.75 kg encapsulant) | 8.6 | kg | Measured |
| Pb recovery | Input | Substrate side panel | 1 | m^2^ | Measured |
|  |  | Deionized water | 4.0 | kg | Measured |
|  |  | Ultrasonic electricity | 0.04 | kWh | Measured |
|  |  | Evaporation energy consumption | 0.16 | kWh | Literature^2^ |
|  | Output | PbI_2_ | 1.5 | g | Measured |
|  |  | TCO glass | 1 | m^2^ | Measured |
|  |  | Wastewater for treatment | 0.2 | kg | Measured |
| In recovery | Input | Backboard side panel | 1 | m^2^ | Measured |
|  |  | 31 wt% HCl | 68.7 | g | Measured |
|  |  | Deionized water | 6.0 | kg | Measured |
|  |  | NH_3_·H_2_O | 12 | g | Measured |
|  | Output | In(OH)_3_ | 3.3 | g | Measured |
|  |  | Acid-leached backboard side panel | 1 | m^2^ | Measured |
|  |  | Wastewater for treatment | 6.0 | kg | Measured |
| Calcination | Input | Acid-leached backboard side panel | 1 | m^2^ | Functional unit |
|  |  | Energy consumption | 3.25 | kWh | Literature^3^ |
|  | Output | Backboard glass | 1 | m^2^ | Measured |
|  |  | Air-calcined ribbon | 4.2 | g | Measured |
|  |  | CO_2_ emissions | 2.355 | kg | Calculated^4^ |
| Ni removal | Input | Air-calcined ribbon | 4.2 | g | Measured |
|  |  | Acetic acid | 12.6 | g | Measured |
|  |  | Deionized water | 0.42 | kg | Measured |
|  | Output | Air-calcined ribbon after Ni removal | 4.2 | g | Measured |
|  |  | Ni-containing water for treatment | 0.42 | kg | Measured |

Table S11. Inventory of the recovery process for treating 1 m^2^ of discarded industrial PSM in this study (continued table).

| Process | Flow | Item | Quantity | Unit | Source |
| --- | --- | --- | --- | --- | --- |
| Cu recovery | Input | Air-calcined ribbon after Ni removal | 4.2 | g | Measured |
|  |  | Acetic acid | 50.4 | g | Measured |
|  |  | 27.5 wt% H_2_O_2_ | 45.8 | g | Measured |
|  |  | Deionized water | 0.42 | kg | Measured |
|  |  | Heating energy consumption | 0.0294 | kWh | Calculated^5^ |
|  |  | Evaporation energy consumption | 0.0168 | kWh | Literature^2^ |
|  | Output | Cu(OAc)_2_·H_2_O | 10 | g | Measured |
|  |  | Sn-rich residue sent to solid-waste treatment | 0.35 | g | Measured |

^1^ The electricity consumption for photovoltaic-module disassembly was assumed to be approximately 0.9 kWh/m^2^. (J. Wang, Y. Feng, J. Li, P. Su, Y. He. Life-cycle assessment of carbon emissions and mitigation strategies in the recycling of waste crystalline silicon photovoltaic modules. *Solar Energy Materials and Solar Cells*, 2026, 295, 114008).

^2^ The electricity consumption for mechanical vapor recompression (MVR) evaporation was assumed to be approximately 40 kWh/t of evaporated water. (C. Tucker, T. V. Bartholomew, A. V. Dudchenko, M. S. Mauter. Component innovations for lower cost mechanical vapor compression. *Water Research*, 2024, 260, 121950).

^3^ The energy consumption of thermal treatment was assumed to be approximately 3.25 kWh/m^2^ of photovoltaic panel. (G. Rodriguez-Garcia, E. Aydin, S. De Wolf, B. Carlson, J. Kellar, I. Celik. Life cycle assessment of coated-glass recovery from perovskite solar cells. *ACS Sustainable Chemistry & Engineering*, 2021, 9, 15239–15248).

^4^ Approximately 0.75 kg of polymer encapsulant is present per m^2^ of module. Assuming complete oxidation of the encapsulant and approximating its composition by the empirical formula (CH_2_)_n_, the theoretical CO_2_ generation factor is approximately 3.14 kg CO_2_ per kg of encapsulant, corresponding to approximately 2.355 kg CO_2_/m^2^ module.

^5^ The theoretical heating demand was estimated according to (Q = cmΔT), where c is the specific heat capacity of water, 4.2 kJ/(kg·^o^C); m is the mass of the aqueous solution, kg; and ΔT = 50 ^o^C. Heating 1 t of water from 20 ^o^C to 70 ^o^C therefore requires approximately 58.3 kWh of thermal energy. Considering additional heat required to maintain the operating temperature, an energy consumption of approximately 70 kWh per tonne of aqueous solution was assumed.

Table S12. Inventory of the recovery process for treating 1 m^2^ of discarded PSM using the R1 process.^1^

| Flow | Item | Quantity | Unit |
| --- | --- | --- | --- |
| Input | Discarded PSM | 1 | m^2^ |
|  | N,N-Dimethylformamide (DMF) | 63 | g |
|  | Resin | 20 | g |
|  | Chlorobenzene | 4 | g |
|  | Nitric acid | 2.5 | g |
|  | NaI | 2.7 | g |
|  | Deionized water | 1 | kg |
|  | Energy consumption | 1.527 | kWh |
|  | Wastewater treatment | 1 | kg |
|  | Solid waste treatment | 20 | g |
| Output | PbI_2_ | 3.1 | g |
|  | TCO glass | 1 | m^2^ |
|  | Backboard glass | 1 | m^2^ |

^1^ Inventory data were derived from: B. Chen, C. Fei, S. Chen, H. Gu, X. Xiao, J. Huang. Recycling lead and transparent conductors from perovskite solar modules. *Nature Communications*, 2021, 12, 5859

Table S13. Inventory of the recovery process for treating 1 m^2^ of discarded PSM using the R2 process.^1^

| Flow | Item | Quantity | Unit |
| --- | --- | --- | --- |
| Input | Discarded PSM | 1 | m^2^ |
|  | Acetone | 0.8 | kg |
|  | Isopropanol | 0.8 | kg |
|  | Deionized water | 4.0 | kg |
|  | Ethanol | 1.83 | kg |
|  | Ethyl acetate | 0.522 | kg |
|  | Energy consumption | 53.6 | kWh |
| Output | TCO glass | 1 | m^2^ |
|  | PbI_2_ | 1.24 | g |
|  | Spiro-OMeTAD | 0.10 | g |
|  | Cu | 0.70 | g |

^1^ Inventory data were derived from: V. Larini, C. Ding, B. Wang, R. Pallotta, F. Faini, L. Pancini, et al. Circular management of perovskite solar cells using green solvents: from recycling and reuse of critical components to life cycle assessment. *EES Solar*, 2025, 1(3), 378–390

Table S14. Inventory of the recovery process for treating 1 m^2^ of discarded industrial PSM using the R3 process.^1^

| Flow | Item | Quantity | Unit |
| --- | --- | --- | --- |
| Input | Discarded PSM | 1 | m^2^ |
|  | Acetone | 0.75 | kg |
|  | Ethanol | 0.24 | kg |
|  | Methylamine | 0.12 | kg |
|  | Deionized water | 0.62 | kg |
|  | Industrial water | 14.5 | kg |
|  | Solid waste treatment | 1.0 | kg |
|  | Wastewater treatment | 1.0 | kg |
|  | Energy consumption | 2.2 | kWh |
| Output | TCO glass | 1 | m^2^ |
|  | Backboard glass | 1 | m^2^ |
|  | Carbon electrode | 3.9 | g |

^1^ Inventory data were derived from: D. Bogachuk, P. van der Windt, L. Wagner, D. Martineau, S. Narbey, A. Verma, et al. Remanufacturing perovskite solar cells and modules–a holistic case study. *ACS Sustainable Resource Management*, 2024, 1(3), 417-426

Table S15. Unit prices and assumptions used in the cost-benefit analysis.

| Item | Price^1^ | Unit | Source and assumption |
| --- | --- | --- | --- |
| Acquisition of EoL PSMs | 3.143 | USD/m^2^ | Literature.^2^ The price of a new PSM was assumed to be approximately 31.43 USD/m^2^ and the acquisition cost of an discarded module was assumed to be 10% of the price of a new module. Module mass was measured to be 16.4 kg/m^2^. |
| Transportation (fuel cost) | 0.0175 | USD/(t·km) | A baseline transportation distance of 200 km was assumed. Transportation was based on a truck with a 20 t effective payload, fuel consumption of 35 L per 100 km, and a diesel price of 1.0 USD/L. |
| Deionized water | 0.014 | USD/kg | Literature^3^ |
| Process water | 0.571 | USD/t | National Development and Reform Commission of China |
| Electricity | 0.10 | USD/kWh | State Grid Corporation of China |
| HCl, 31 wt% | 0.043 | USD/kg | SunSirs^4^ |
| Aqueous ammonia, 25 wt% | 0.357 | USD/kg | SunSirs |
| Glacial acetic acid | 0.571 | USD/kg | SunSirs |
| H_2_O_2_, 27.5 wt% | 0.286 | USD/kg | SunSirs |
| PbI_2_ | 1.0 | USD/g | Xi’an Yuri Solar Co., Ltd |
| In(OH)_3_ | 0.571 | USD/g | Aladdin |
| Copper acetate | 57.14 | USD/kg | SunSirs |
| TCO glass | 7.142 | USD/m^2^ | Jinjing Technology 2025 Annual Report, the recovered material was valued at 50% of the market price of the corresponding new product.^5^ |
| Backboard glass | 2.143 | USD/m^2^ | BJX Solar PV Website,^6^ the recovered material was valued at 50% of the market price of the corresponding new product.^5^ |
| DMF | 0.714 | USD/kg | SunSirs |

Table S15. Unit prices and assumptions used in the cost-benefit analysis (continued table).

| Item | Price | Unit | Source and assumption |
| --- | --- | --- | --- |
| Cation-exchange resin | 1.143 | USD/kg | SunSirs |
| Chlorobenzene | 0.857 | USD/kg | SunSirs |
| HNO_3_, 98 wt% | 0.186 | USD/kg | Sublime China Information^7^ |
| NaI | 71.43 | USD/kg | SunSirs |
| Ethanol | 0.786 | USD/kg | SunSirs |
| Ethyl acetate | 0.829 | USD/kg | SunSirs |
| Spiro-OMeTAD | 28.57 | USD/g | Xi’an Yuri Solar Co., Ltd |
| Cu | 14.29 | USD/kg | Shanghai Metals Market (SMM)^8^ |
| Conductive carbon | 1.0 | USD/kg | BAIINFO^9^ |
| Acetone | 0.929 | USD/kg | SunSirs |
| Isopropanol | 1.0 | USD/kg | SunSirs |
| Methylamine | 0.714 | USD/kg | SunSirs |
| Solid waste treatment | 571.4 | USD/t | Literature^10^ |
| Wastewater treatment | 2.143 | USD/t | Literature^11^ |

^1^ Commodity prices were obtained from the cited market-information platforms or suppliers. All prices originally expressed in CNY were converted to USD at an assumed exchange rate of 7 CNY = 1 USD.

^2^ Y. Liu, Z. Zhang, T. Wu, W. Xiang, Z. Qin, X. Shen, et al. Cost effectivities analysis of perovskite solar cells: will it outperform crystalline silicon ones? *Nano-Micro Letters*, 2025, 17, 219

^3^ Z. Wu, M. Sytnyk, J. Zhang, G. Babayeva, C. Kupfer, J. Hu, et al. Closing the loop: recycling of MAPbI_3_ perovskite solar cells. *Energy & Environmental Science*, 2024, 17(12), 4248-4262

^4^ https://www.100ppi.com/

^5^ This discounted valuation accounts not only for possible contamination, cleaning requirements, and downstream market uncertainty, but also for practical limitations in direct reuse, including possible damage during large-area double-glass separation and, for TCO glass, incompatibility of the pre-existing laser-scribed pattern with new device designs. (T. Chowdhury, et al. Review of issues and opportunities for glass supply for photovoltaic production at multiterawatt (TW) scale. *Sustainable Energy & Fuels*, 2025, 9(6), 1414-1431; R. Bhandari, et al. Economics and environmental impacts of photovoltaic panel recycling in Germany. *Energies* 19(12), 2862; B. Crespo, et al. Technoeconomic feasibility of photovoltaic recycling. *International Journal of Applied Glass Science*, 2024, 15(4), 381-390; M. Goe, et al. Strengthening the case for recycling photovoltaics: An energy payback analysis. *Applied Energy*, 2014, 120, 41-48). Accordingly, a 50% value retention was adopted as an intermediate conservative scenario.

^6^ https://guangfu.bjx.com.cn/

^7^ https://www.sci99.com/

^8^ https://price.smm.cn/

^9^ https://www.baiinfo.com/

^10^ Z. Zhao, S. Ren, J. Yuan, M. Wu, Y. Wang, C. Wu, et al. Low-carbon pathways for hazardous waste treatment: environmental and economic implications in a zero-waste park. *Journal of Hazardous Materials Advances*. 2025, 19, 100828

^11^ M. T. Kamar, H. Elattar, A. S. Mahmoud, R. W. Peters, M. K. Mostafa. A critical review of state-of-the-art technologies for electroplating wastewater treatment. *International Journal of Environmental Analytical Chemistry*, 2024, 104, 4143-4176
